# First record of the family Sphyrapodidae Guţu, 1980 (Crustacea: Peracarida: Apseudomorpha) with the description of a new species of *Sphyrapus* from the Colombian Caribbean

**DOI:** 10.7717/peerj.3947

**Published:** 2017-10-19

**Authors:** Andrés G. Morales-Núñez, Catalina Morales-Ruiz, Néstor E. Ardila

**Affiliations:** 1 NSF—CREST Center for the Integrated Study of Coastal Ecosystem Processes and Dynamics in the Mid-Atlantic Region (CISCEP), Department of Natural Sciences, University of Maryland Eastern Shore, Princess Anne, MD, USA; 2 División de Biología Marina, ECOMAR Consultoría Ambiental, Bogotá, Colombia

**Keywords:** Tanaidacea, Colombia, *Kudinopasternakia*, *Sphyrapus caribensis*, Caribbean, Sphyrapodidae, Deep-sea

## Abstract

A new sphyrapodid tanaidacean, *Sphyrapus caribensis* sp. nov. is described and a new record of *Kudinopasternakia siegi* is reported for the Colombian Caribbean based on samples collected during cruises in 2014–2015. The new species appears to be most closely related to the northeast Atlantic species, *Sphyrapus malleolus. Sphyrapus caribensis* can be distinguished from *Sphyrapus malleolus* by a combination of characters, including the maxillipedal basis without long distal seta, the number of setae on the distoventral margin of pereopods 1 and 2, and the number of plumose seta on the pleopod basis. A key for the separation of *Sphyrapus* species is presented.

## Introduction

Members of the order Tanaidacea Dana, 1849 are inhabitants of brackish and marine environments at all latitudes throughout the world (Larsen, 2005). In the Caribbean region, tanaidaceans are poorly studied and hence information their taxonomy, systematics, and ecology is scarce. During the last two decades, however, the number of new species-descriptions and records of tanaidaceans in the northern Caribbean has increased (Hansknecht, Heard & Martin, 2001; Guţu & Heard, 2002; Hansknecht & Santos, 2008; Guţu, 2009; Morales-Núñez, 2010; Morales-Núñez & Heard, 2013, 2014; Jarquín-González, 2016).

Information on this group in Colombia, which faces to the southern Caribbean and the Pacific, is limited to two taxonomic studies from shallow waters along the Pacific coast: (1) Menzies (1953) described *Apseudomorpha veleronis* (Menzies, 1953) based on samples collected in Octavia Bay (Chocó Department); and (2) Guţu & Ramos (1995) recorded *Sinelobus stanfordi* (Richardson, 1901) in the “Ensenada de Utría” (Chocó Department), and also described two new species *Discapseudes colombiensis* Guţu & Ramos, 1995 from Buenaventura Bay and *Aparatanais denticulatus* (Guţu & Ramos, 1995) from Gorgonilla Island.

The family Sphyrapodidae Guţu, 1980 is a small group of apseudomorphan tanaidaceans that has been reported from coastal to deep waters (Guţu & Heard, 2002; Heard, Hansknecht & Larsen, 2004; Larsen, 2005; Kakui, Kajihara & Mawatari, 2007; Bamber & Marshall, 2013), and is characterized by the reduction or loss of the antennal squama, large size of the pereopod-1, and lack of spiniform apophyses on the carapace, eye-lobes, and pereopod coxa. Currently, the family is divided into two subfamilies (Pseudosphyrapodinae Guţu, 1980 and Sphyrapodinae Guţu, 1980), seven genera and 29 species (Anderson, 2014).

Recently, explorations along the outer shelf, continental slope, and continental margin of the Caribbean coast of Colombia during cruises in 2014–2015 resulted in the collection of specimens of *Kudinopasternakia siegi* (Viskup & Heard, 1989) and specimens of an undescribed species of the genus *Sphyrapus* Norman, 1882. In this paper, we reported for the first time the family Sphyrapodidae in the Colombian Caribbean, and provide a description of a new species of *Sphyrapus*.

## Materials and Methods

**Figure 1:**
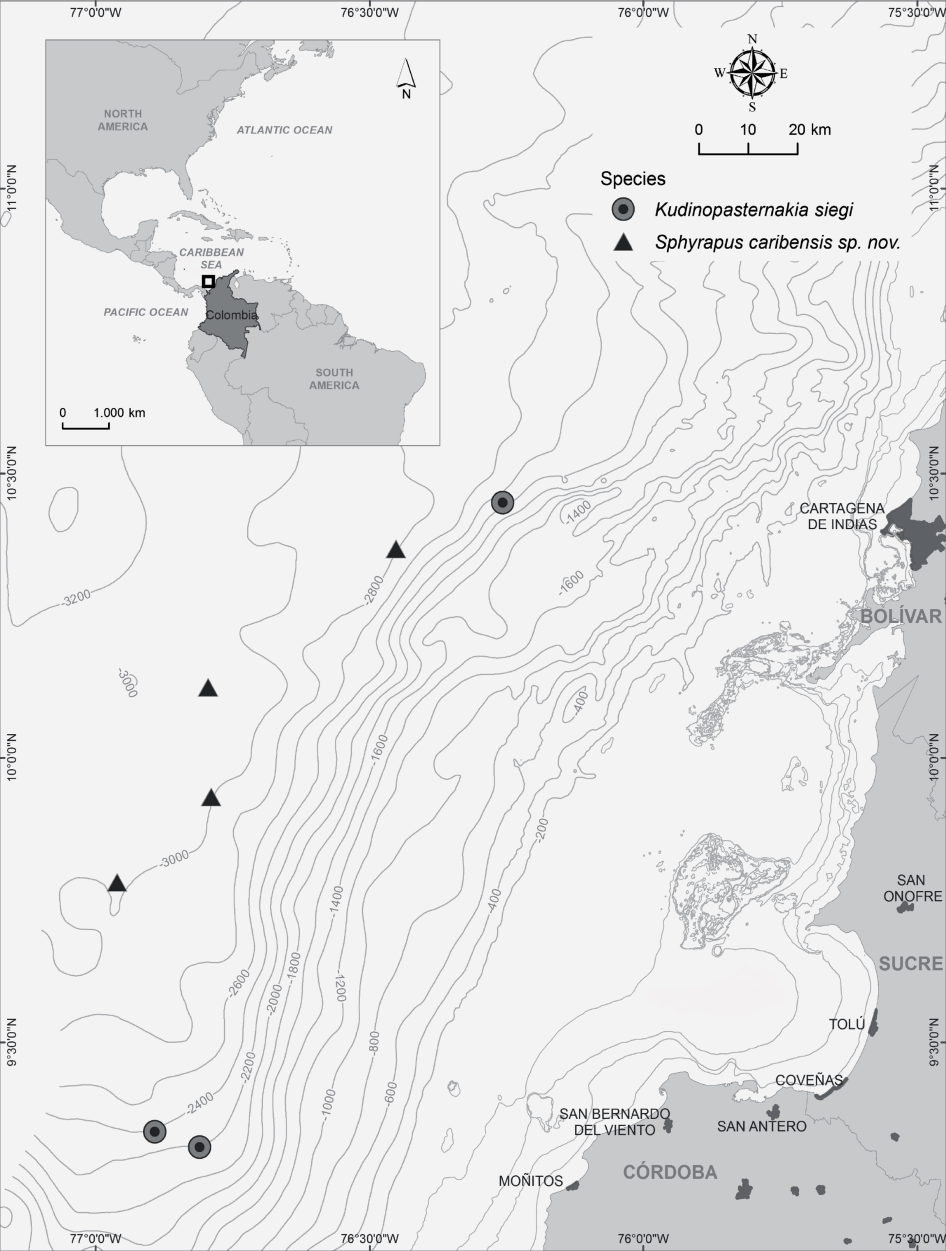
Map of study area, indicating the sampling stations where *Kudinopasternakia siegi* and *Sphyrapus caribensis* sp. nov. were found.

Samples were collected using a box corer of 0.25 m^2^ during cruises, aboard the R/V *Proteus* and R/V *Don Rodrigo-B*, working off the southwestern Caribbean Sea of Colombia at depths of 176 to 3,094 m (Fig. 1). Tanaidaceans were sorted, fixed in 6% formalin, and subsequently stored in 70% ethanol. Collection permits were granted by the National Authority of Environmental Licenses—ANLA (FNA, ANLA No. 0723 de 2012; PAC, ANLA No. 0880 de 2014; COL5, ANLA No. 0440 de 2015).

Sphyrapodid specimens were dissected under a stereomicroscope Olympus ZS-16. Appendages were mounted on glass slides in glycerine and observed with an Olympus BX41 microscope. Drawings were made with a *camera lucida* and illustrations were prepared with Adobe Illustrator CC and Photoshop CC. Photographs were taken using an Olympus DP73 digital camera mounted on a stereomicroscope and all specimens were measured with CellSens Dimension 1.11 Imaging Software (Olympus, Tokyo, Japan). Maps were created using ArcGIS 10.4.1 software (University of Maryland Eastern Shore (UMES)).

Topotypic specimens of *Kudinopasternakia siegi* (Viskup & Heard, 1989) collected from the Gulf of Mexico (GoM) were made available to us by Dr. Richard W. Heard (personal collection).

All measurements were taken in millimeters (mm). Total body length (TL) was measured from the tip of the rostrum to the tip of the pleotelson. We defined the first fused or unfused aesthetasc-bearing article on the male antennule as flagellum article-1. Terminology generally follows that of Larsen (2003), with the following exception: the term “serratopinnate” is here applied to those setae having the main body serrate and the apex pinnate (Santos, 2014). Type material and specimens of previously known species have been deposited at the “Centro de Colecciones Biológicas, Universidad del Magdalena (CBUMAG),” Santa Marta, Colombia, and National Museum of Natural History, Smithsonian Institution, Washington, DC (USNM).

The electronic version of this article in portable document format will represent a published work according to the International Commission on Zoological Nomenclature (ICZN), and hence the new names contained in the electronic version are effectively published under that Code from the electronic edition alone. This published work and the nomenclatural acts it contains have been registered in ZooBank, the online registration system for the ICZN. The ZooBank LSIDs (Life Science Identifiers) can be resolved and the associated information viewed through any standard web browser by appending the LSID to the prefix http://zoobank.org/. The LSID for this publication is: [urn:lsid:zoobank.org:pub:2ADB8B48-19CB-4DF0-8FBD-CF9DA78E779D]. The online version of this work is archived and available from the following digital repositories: PeerJ, PubMed Central and CLOCKSS.

### Systematics

**Order Tanaidacea Dana, 1849**
**Suborder Apseudomorpha Sieg, 1980**
**Superfamily Apseudoidea Leach, 1814**
**Family Sphyrapodidae Guţu, 1980**
**Subfamily Pseudosphyrapodinae Guţu, 1980**
**Genus *Kudinopasternakia*Guţu, 1991**

**Type-species:** *Pseudosphyrapus larisae* Guţu, 1989

**Diagnosis:** See Guţu (1991: new genus and diagnosis); Larsen (2005: modified diagnosis and remarks).

**Composition (10 species):** *Kudinopasternakia amazonica* Santos, 2007; *Kudinopasternakia balanorostrata* Kakui, Kajihara & Mawatari, 2007; *Kudinopasternakia bispinosa* Guţu & Heard, 2002; *Kudinopasternakia brasiliensis* Santos, 2007; *Kudinopasternakia dispar* (Lang, 1968); *Kudinopasternakia falconae* Santos, 2014; *Kudinopasternakia larisae* (Guţu, 1989); *Kudinopasternakia serejae* Santos, 2014; *Kudinopasternakia siegi* (Viskup & Heard, 1989); *Kudinopasternakia trispinosa* Santos, 2007.

**Figure 2:**
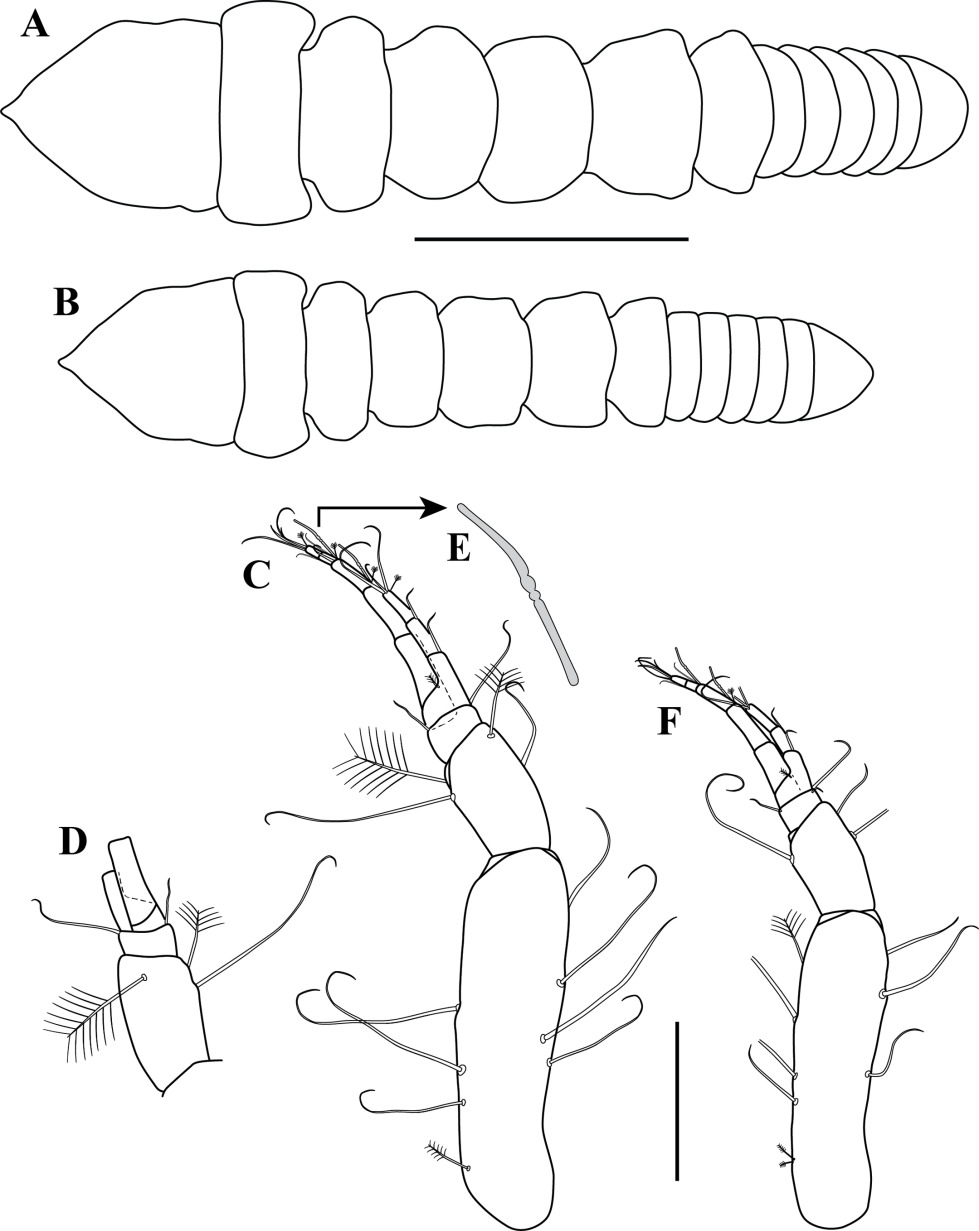
*Kudinopasternakia siegi* (Viskup & Heard, 1989). (A) Female with oostegites, TL 8.2 mm: dorsal view, from Colombian Caribbean; (B) female with oostegites, TL 6.3 mm, dorsal view, from Gulf of Mexico; (C) antennule; (D) opposite view of antennal article-2 to article-4 (common article); (E) enlargement of aesthetasc; (F) antennule, specimen from Gulf of Mexico. Scale bar = 2.0 mm for A, B; scale bar = 1.0 mm for C, D, F.

**Figure 3:**
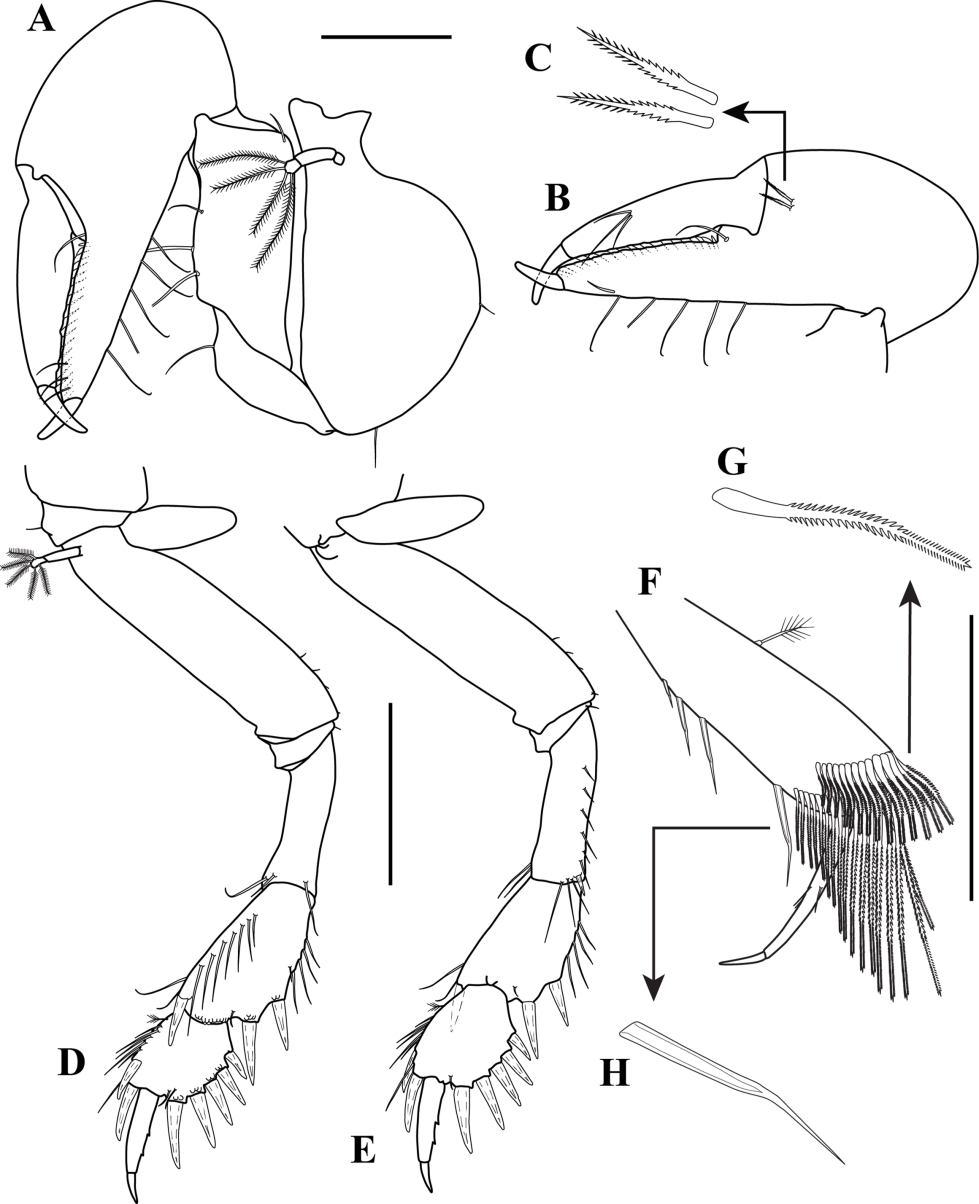
*Kudinopasternakia siegi* (Viskup & Heard, 1989) from Colombian Caribbean, female with oostegites, TL 8.2 mm. (A) Cheliped, outer view; (B) chela, inner view; (C) enlargement of serratopinnate seta; (D) pereopod-1, outer view; (E) pereopod-1, inner view; (F) propodus and dactylus of pereopod-4; (G) enlargement of serratopinnate seta; (H) enlargement of spiniform seta. Scale bar = 0.5 mm A–E.

**Figure 4:**
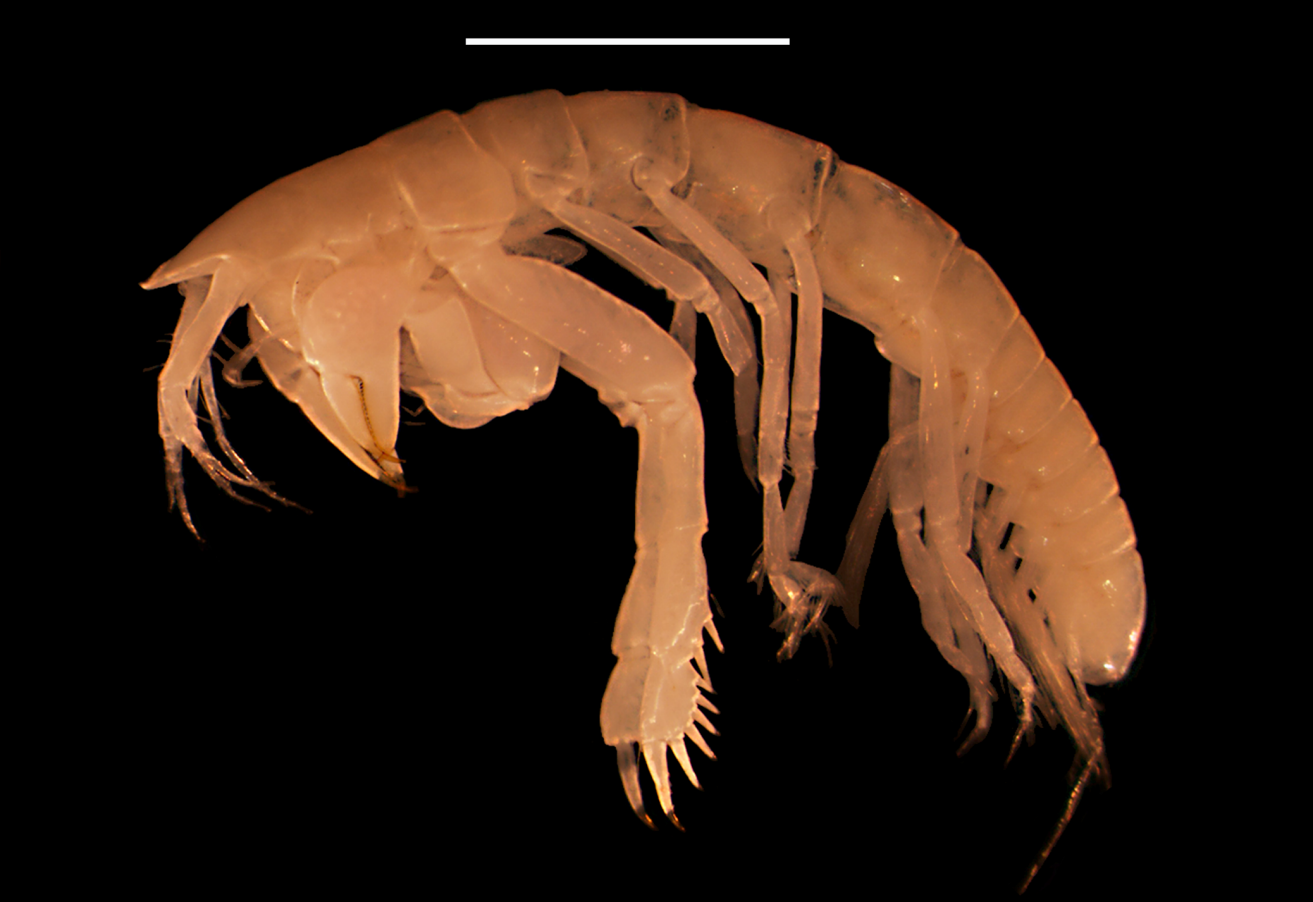
Digital image of *Kudinopasternakia siegi*, female: lateral view of habitus. Scale bar = 2.0 mm.

***Kudinopasternakia siegi* (Viskup & Heard, 1989)**
(Figs. 2–4)

*Pseudosphyrapus siegi* (Viskup & Heard, 1989: new species and diagnosis); *Kudinopasternakia siegi* (Guţu & Heard, 2002: new combination); Larsen (2005: modified diagnosis and remarks).

**Amended diagnosis:** *Female. Rostrum* lateral margins straight or slightly convex, pointed. Antennular outer flagellum with five articles. *Antenna* with nine articles. *Epimera* rounded. *Cheliped* with carpus lacking process on anterior margin; propodus with two serratopinnate setae on medial inner margin near articulation with dactylus. *Pereopod-1* carpus and propodus with two and five spiniform setae on ventral margin, respectively; exopod with 4–5 plumose setae. Pereopod-4 propodus with 4–5 simple setae on ventral margin.

**Material examined:** One ♀ with oostegites (partially dissected), (CBUMAG: MAC: 00003), TL 8.2 mm, Station (Stn) E06PAC (9°18′53.84″N–76°48′35.95″W), depth 2,229 m, substrata: “muddy bottom,” 26 July 2014; one non-ovigerous ♀ (partially dissected), (CBUMAG: MAC: 00004), TL 5.4 mm, Stn E07Col5c (9°20′31.73″N–76°53′30.67″W), depth 2,380 m, substrata: “muddy bottom,” 03 May 2015; one manca, (CBUMAG: MAC: 00005), TL 3.3 mm, Stn E06FNA (10°26′55.54″N–76°15′25.29″W), depth 2,206 m, substrata: “muddy bottom,” 23 July 2014.

**Additional material from Gulf of Mexico (GoM):** One non-ovigerous ♀ (partially dissected), (USNM 1437641), TL 6.3 mm, Stn NB3(5) (26°32′30.39″N–91°45′51.32″W), depth 1,875 m, 8 May 2000; One subadult ♀, TL 4.80 mm; One non-ovigerous ♀, TL 6.4 mm.

***Partial description of a female with oostegites (CBUMAG 00003) of Kudinopasternakia siegi from Colombian Caribbean:***

*Body* (Fig. 2A) slender, about 5.2 times as long as wide.
*Cephalothorax* (Fig. 2A) about 20% of TL, shorter than combined lengths of pereonites 1–3, length 1.2 times longer than wide, carapace asetose; rostrum small and triangular; eyes-lobes present (small) without visual elements.
*Pereon* (Fig. 2A) about 60% of TL, six free pereonites, all pereonites wider than long; pereonite-1 rectangular, wider than carapace and other pereonites; pereonites 4–5 longest; pereonite-6 shortest.
*Pleon* (Fig. 2A) about 15% of TL, combined lengths of pleonites 1–5 shorter than pereonites 5–6 combined; all pleonites sub-equal, wider than long, laterally rounded, bearing pleopods.
*Pleotelson* (Fig. 2A) about 5% of TL, wider than long, about same length as pleonites 4–5 combined.
*Antennule* (Figs. 2C–2E) peduncle with four articles; article-1, 3.6 times as long as wide, inner margin with four long simple setae, outer margin with one broom-seta proximally and three long simple setae; article-2, 1.6 times as long as wide, inner margin and outer margins each with one long simple seta and one broom-seta; article-3, broader than long, inner margin with long simple seta distally, outer margin with simple setae distally; article-4 (common article) (Figs. 2C and 2D), with small broom-seta at insertion with outer flagellum, and one simple seta at insertion of inner flagellum. Inner flagellum biarticulate; article-1, with one simple seta distally; article-2, with one broom-seta and three simple setae of varying lengths. Outer flagellum with five articles; article-1 asetose; article-2, with broom-seta and one aesthetasc; articles 3, with two simple setae and one aesthetasc; article-4, with one simple seta, article-5, with four simple setae of varying lengths and one broom-seta.
*Cheliped* (Figs. 3A–3C) with basis, 1.7 times as long as wide, bearing short simple seta on mid-ventral margin and long simple seta on sub-distal ventral margin. Merus sub-rectangular, with sub-distal simple seta on ventral margin. Carpus, 1.8 times as long as wide, longer than merus, with distodorsal simple seta; with four simple setae on ventral margin. Propodus, with one simple seta at dactylus insertion with dactylus, with two serratopinnate setae on medial inner margin near articulation with dactylus (Figs. 3B–3C); fixed finger with three simple setae on outer incisive margin and row of lamellae on cutting surface; with five ventral setae, unguis robust. Dactylus as long as fixed finger, with three sub-distal simple setae and row of lamellae on cutting surface, claw robust. Exopod with three articles, article-3 bearing four plumose setae.
*Pereopod*-*1* (Figs. 3D, 3E and 4) distinctly larger and longer than other five pereopods (Fig. 4). Coxa wider than long with one simple setae on mid-dorsal margin. Basis stout, 3.5 times as long as wide; sub-distal ventral margin with four simple setae. Ischium wider than long, with simple seta on mid-ventral margin. Merus, 3.2 times as long as wide; outer margin with two distodorsal simple setae and one distoventral simple seta; inner margin with nine ventral simple setae, and one distoventral spiniform seta (Fig. 3E). Carpus, 1.9 times as long as wide; dorsal margin with a row of nine simple setae and one distal spiniform seta; ventral margin with four long, two outer (one on mid-margin and one on distal margin) short, and one inner distal short (Fig. 3D) simple setae, and two strong spiniform setae. Propodus, 1.1 times as long as wide; dorsal margin with broom-seta, six simple setae, and two distal spiniform setae; ventral margin with five strong spiniform setae becoming longer distally, and two outer distal and one inner distal (Fig. 3D) short simple setae. Dactylus together with unguis longer than propodus; dactylus longer than unguis with two spines on ventral margin. Exopod with five plumose setae.
*Pereopod-4* (partial illustrated) basis, 5.6 times as long as wide; dorsal margin with three broom-setae; ventral margin with mid-broom-seta and sub-distal simple seta. Ischium wider than long with simple setae on mid-ventral margin. Merus, 1.5 times as long as wide, with two simple setae on sub-distal ventral margin. Carpus longer than merus or propodus, 2.8 times as long as wide; outer margin with a row of nine simple setae; ventral margin with one simple setae. Propodus (Fig. 3F) 3.0 times as long as wide; dorsal margin with broom-seta; distally with two crown of ∼14–16 and ∼20–22 serratopinnate setae (Fig. 3G), respectively; ventral margin with four simple setae (Fig. 3H). Dactylus (Fig. 3F) together with unguis shorter than propodus; dactylus longer than unguis, with mid-outer and two mid-ventral simple setae.

### Ecological notes

Specimens of *Kudinopasternakia siegi* (Fig. 4) were collected from muddy bottoms with a content of mud and clay between 93.3% and 95.6%. Other physicochemical parameters of the surrounding waters include a temperature of 4.1 °C, salinity of 35 ppm, pH of 7.93–8.0, and dissolved oxygen (DO) of 4.7–6.8 mg/L.

***Partial description of a female with oostegites (USNM 1437641) of Kudinopasternakia siegi from Gulf of Mexico:***

*Body* (Fig. 2B) slender, about 5.3 times as long as wide.
*Cephalothorax* (Fig. 2B) about 20% of TL, shorter than combined lengths of pereonites 1–3, length 1.2 times longer than wide, carapace asetose; rostrum small and triangular.
*Pereon* (Fig. 2B) about 55% of TL, six free pereonites, all pereonites wider than long; pereonite-1 rectangular, wider than carapace and pereonites; pereonites 4–5 longest; pereonite-6 shortest.
*Pleon* (Fig. 2B) about 20% of TL; combined length of pleonites 1–5 similar to that of pereonites 5–6 combined; all pleonites sub-equal, wider than long, laterally rounded, bearing pleopods.
*Pleotelson* (Fig. 2B) about 5% of TL; wider than long, slightly longer than pleonites 4–5 combined.
*Antennule* (Fig. 2F) peduncle with four articles; article-1, 3.8 times as long as wide, inner margin with three long simple setae, outer margin with three (two proximally and one sub-distally) broom-setae and three long simple setae; article-2, 1.6 times as long as wide, inner margin with two simple setae, and outer margin with one long simple seta; article-3, broader than long, inner margin with two (one short and one long) simple seta distally, outer margin with simple setae distally; article-4 (common article), with small broom-seta at insertion with outer flagellum, and one simple seta at insertion of inner flagellum. Inner flagellum biarticulate; article-1, with one simple seta distally; article-2, with one broom-seta and three simple setae of varying lengths. Outer flagellum with five articles; article-1 asetose; article-2, with broom-seta and one aesthetasc; article-3, with one simple setae and one aesthetasc; article-4, with one simple seta; article-5, with four simple setae of varying lengths.

Pereopod-4 (not illustrated) propodus distally with two crown of ∼13 and ∼22 serratopinnate setae, respectively; ventral margin with four simple setae.

### Remarks

In the original description of *Kudinopasternakia siegi*, Viskup & Heard (1989) stated that the antennular inner flagellum has three articles and expressed doubt about the number of articles (four?) observed on the antennular outer flagellum of the species. In this study, we have carefully examined several topotypic females of *Kudinopasternakia siegi*, and confirmed the presence of only two articles on the inner flagellum of antennule; Viskup & Heard (1989) might have included the common article in their account (Viskup & Heard, 1989, fig. 2A, p. 110). Moreover, the antennular outer flagellum has five articles, not four as was stated (see Viskup & Heard, 1989, fig. 2A, p. 110).

The Colombian material is conspecific with *Kudinopasternakia siegi* by having (1) rostrum pointed, (2) antennule inner flagellum with two articles, (3) antenna with nine articles, (4) pereopod-1 with propodus having five spiniform setae on ventral margin, (5) pereopod-4 with propodus having four simple setae on ventral margin, and (6) epimera on pleonites rounded.

This is the first time that a member of the subfamily Pseudosphyrapodinae has been reported from Colombian waters and the Caribbean Sea. The genus *Kudinopasternakia* has been previously reported from the Gulf of Mexico (Viskup & Heard, 1989; Guţu & Heard, 2002), Brazil (Santos, 2007, 2014), Japan (Kakui, Kajihara & Mawatari, 2007), Tasman Sea (Lang, 1968), and Indian Ocean (Guţu, 1989). The occurrence of *Kudinopasternakia siegi* in the deep marine waters of Colombia extends the distribution range of the genus *Kudinopasternakia* to the southern area of the Caribbean Sea.

**Subfamily Sphyrapodinae Guţu, 1980**
**Genus *Sphyrapus* Norman, 1882**

**Type-species:** *Sphyrapus malleolus* Norman & Stebbing, 1886

**Diagnosis:** See Norman & Stebbing (1886: new genus and diagnosis), Błażewicz-Paszkowycz, Bamber & Cunha (2011: redescription of *Sphyrapus malleolus*).

**Composition (three species):** *Sphyrapus caribensis* sp. nov.; *Sphyrapus malleolus*; *Sphyrapus meknes* Błażewicz-Paszkowycz, Bamber & Cunha, 2011.

***Sphyrapus caribensis* sp. nov.**
urn:lsid:zoobank.org:act:93557B1D-0BAE-458F-AFCCCB6D07A90BEB
(Figs. 5–17, 19C, 19E, 19G, 19I, and 19K)

**Figure 5:**
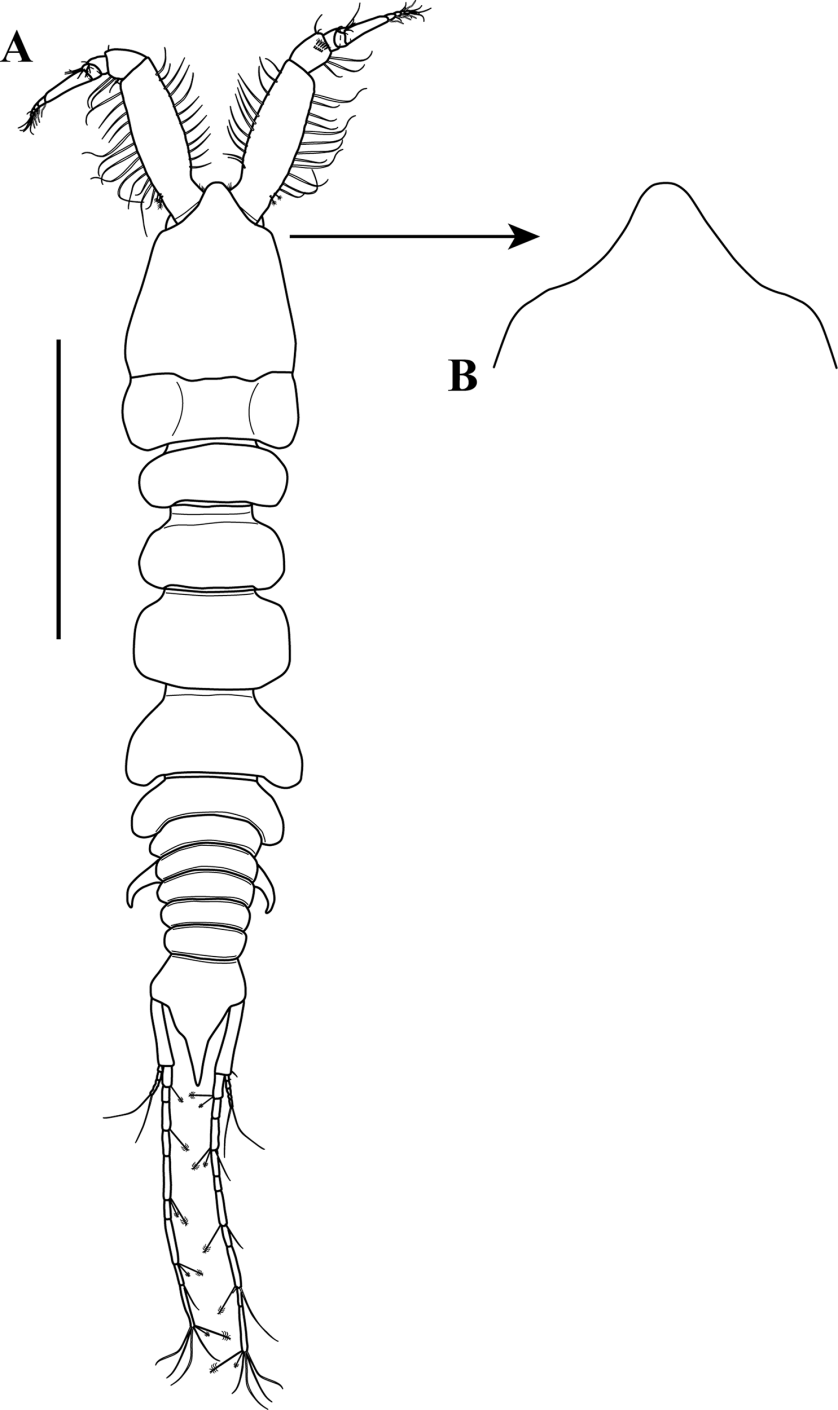
*Sphyrapus caribensis* sp. nov., paratype female. (A) Dorsal view; (B) enlargement of rostrum. Scale bar = 1.0 mm.

**Figure 6:**
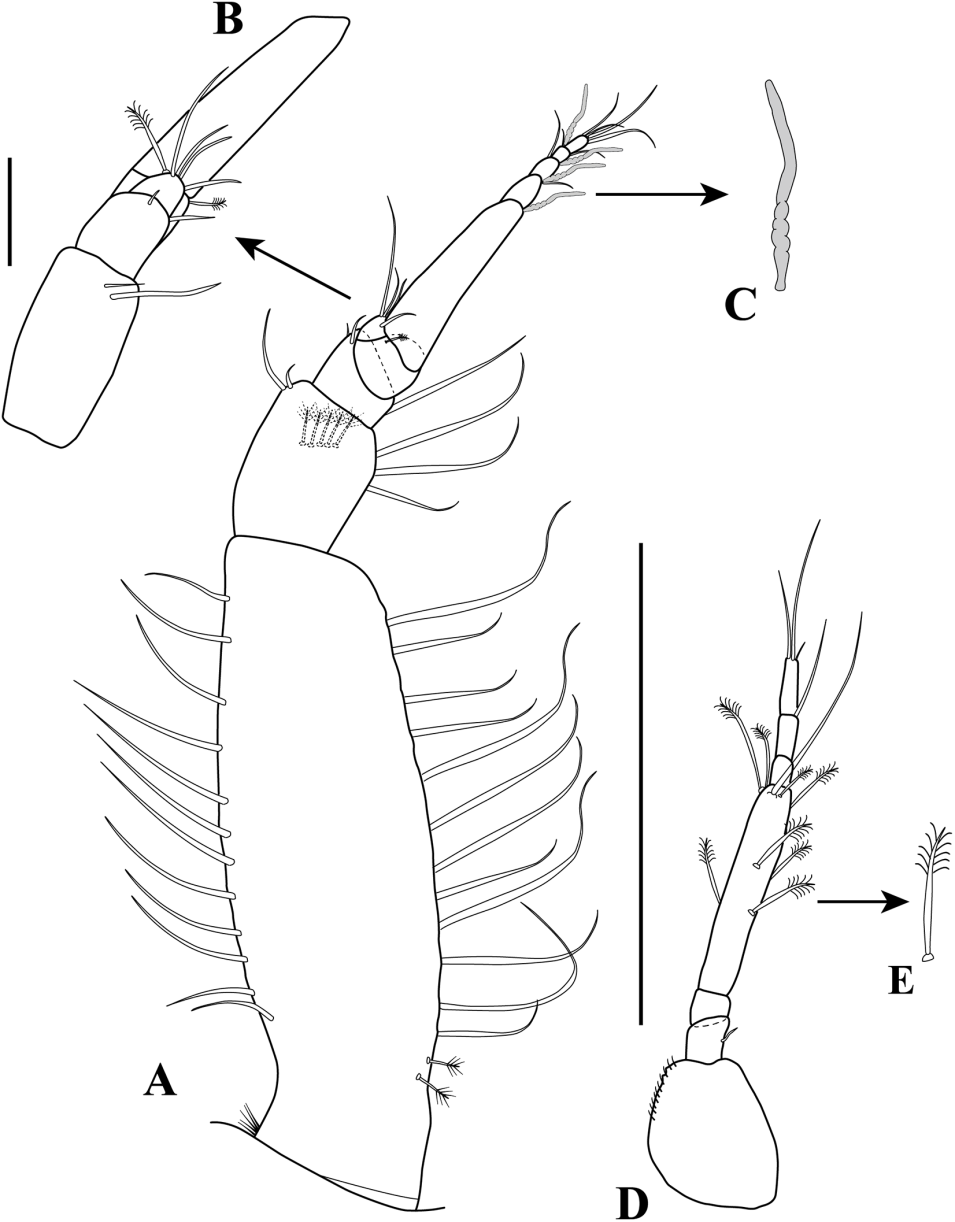
*Sphyrapus caribensis* sp. nov., paratype female. (A) Antennule; (B) enlargement of accessory flagellum; (C) convoluted aesthetasc; (D) antenna; (E) broom-seta. Scale bars = 0.1 mm for A, D.

**Figure 7:**
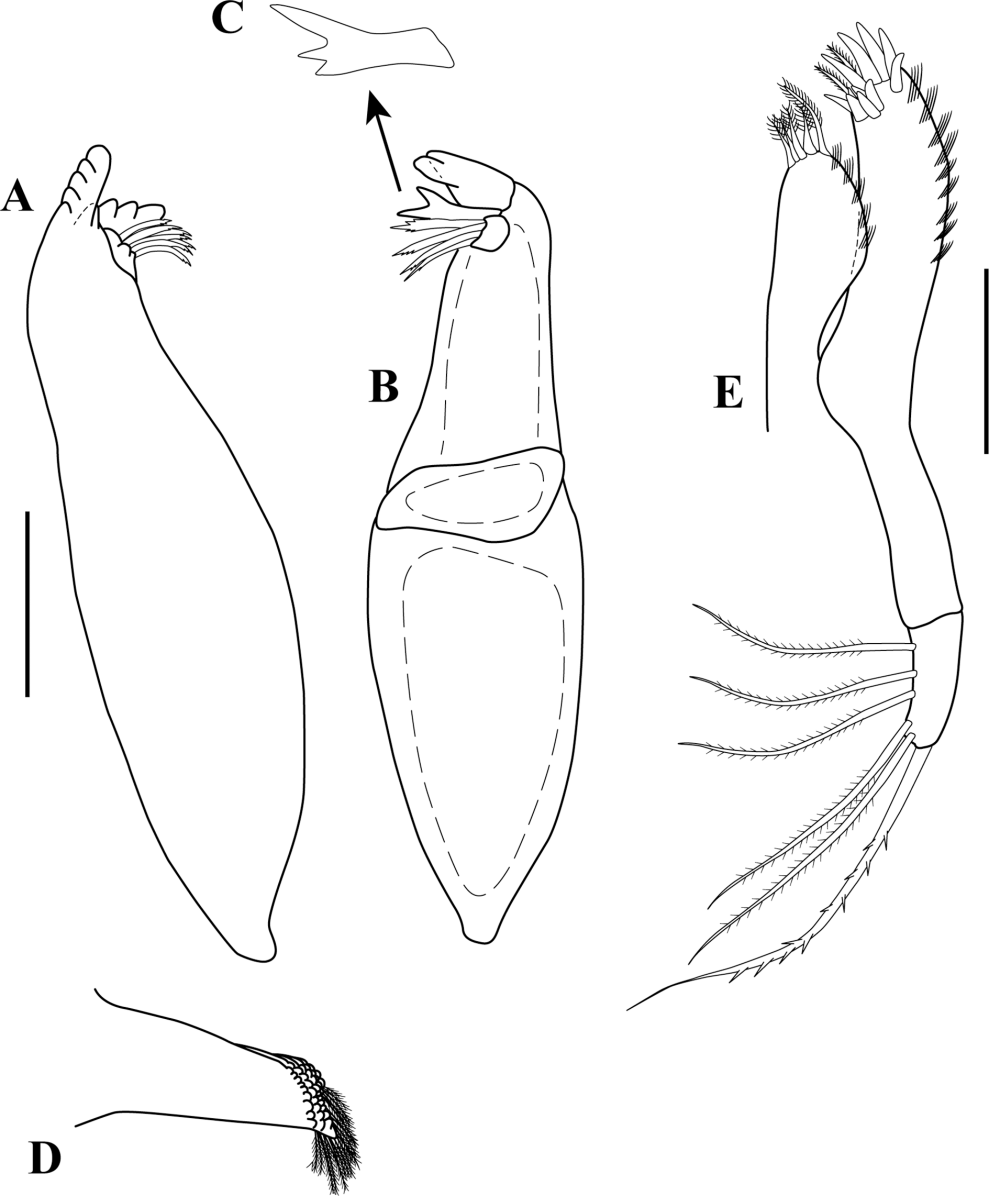
*Sphyrapus caribensis* sp. nov., paratype female. (A) Left mandible; (B) right mandible; (C) enlargement of right lacinia mobilis; (D) left molar process; (E) maxillule. Scale bars = 0.1 mm for A, B, E.

**Figure 8:**
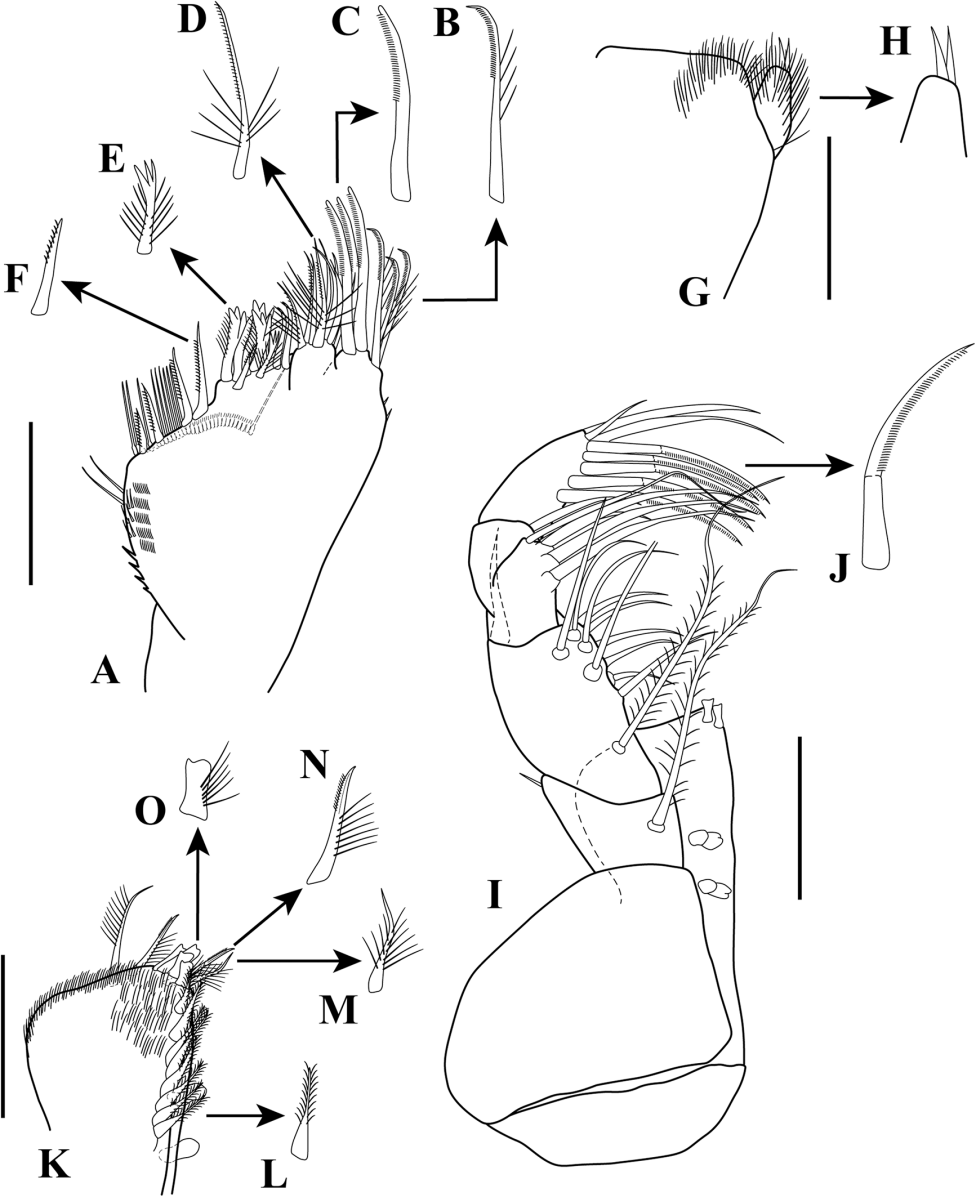
*Sphyrapus caribensis* sp. nov., paratype female. (A) Maxilla; (B) enlargement of finely inner pinnate-outer setulose spiniform seta; (C) enlargement of inner pinnate spiniform seta; (D) enlargement of inner pinnate–plumose spiniform seta; (E) enlargement of trifurcate–plumose spiniform seta; (F) enlargement of short bipinnate seta; (G) labium; (H) enlargement of labium palp; (I) maxilliped; (J) enlargement of inner pinnate spiniform seta; (K) endite; (L) enlargement of basally swollen setulate seta; (M) enlargement of bipinnate spiniform setae; (N) enlargement of inner setulate-outer plumose spiniform seta; (O) enlargement of bidentate or grooved spiniform setae with outer setulose margin. Scale bars = 0.1 mm for A, G, I, K.

**Figure 9:**
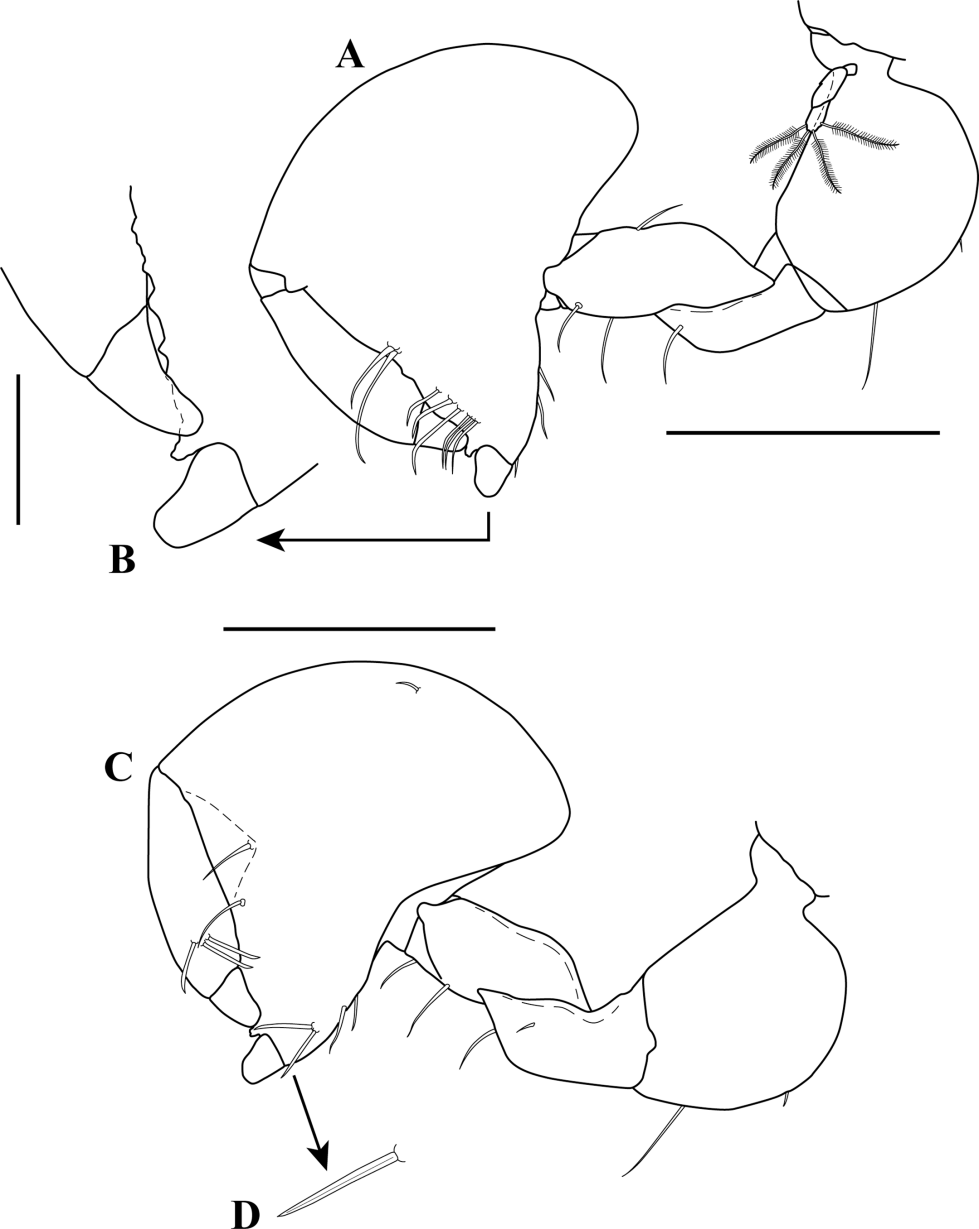
*Sphyrapus caribensis* sp. nov., paratype female. (A) Right cheliped, outer view; (B) enlargement of dorsal margin of fixed finger; (C) right cheliped, inner view; (D) enlargement of spiniform seta. Scale bars = 0.1 mm for A–C.

**Figure 10:**
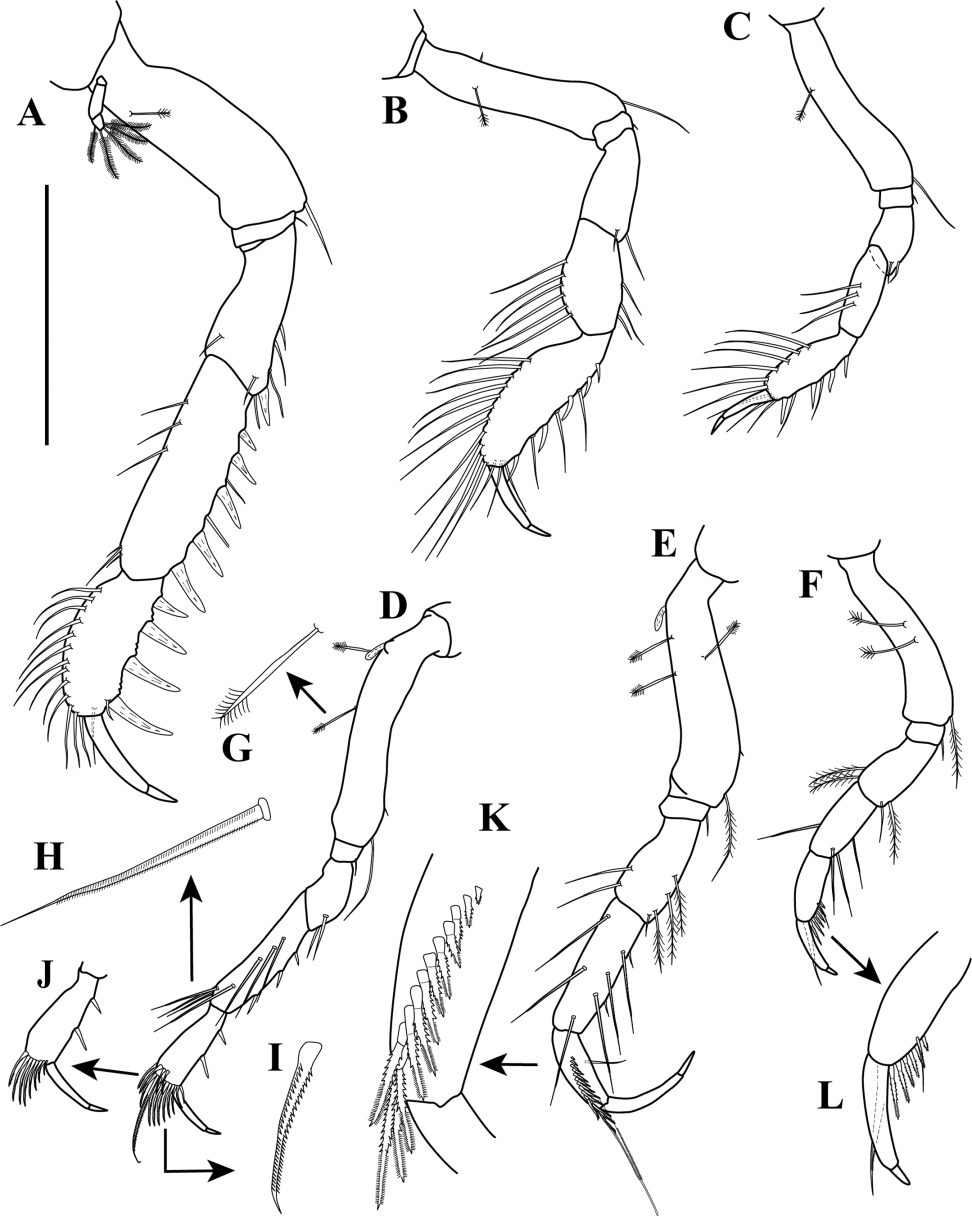
*Sphyrapus caribensis* sp. nov., paratype female. (A) Pereopod-1; (B) pereopod-2; (C) pereopod-3; (D) pereopod-4; (E) pereopod-5: (F) pereopod-6; (G) enlargement of broom-seta; (H) enlargement of bipinnate seta; (I) enlargement of serratopinnate seta; (J) inner view of carpus and dactylus; (K) enlargement of carpus showed the row of 13 short serratopinnate seta; (L) enlargement of carpus and dactylus. Scale bars = 0.1 mm for A–F.

**Figure 11:**
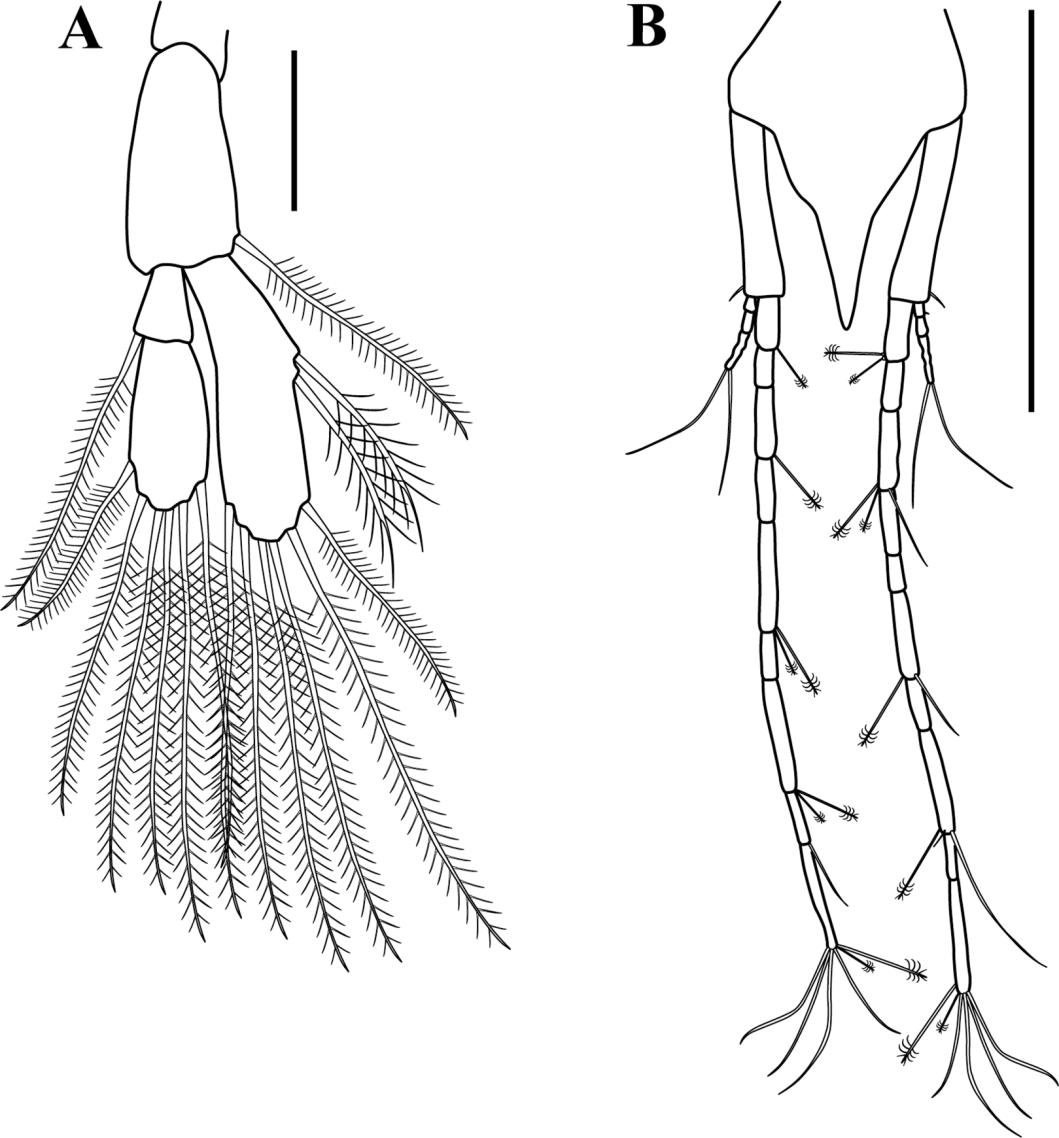
*Sphyrapus caribensis* sp. nov., paratype female. (A) Pleopod; (B) uropod. Scale bars = 0.1 mm.

**Figure 12:**
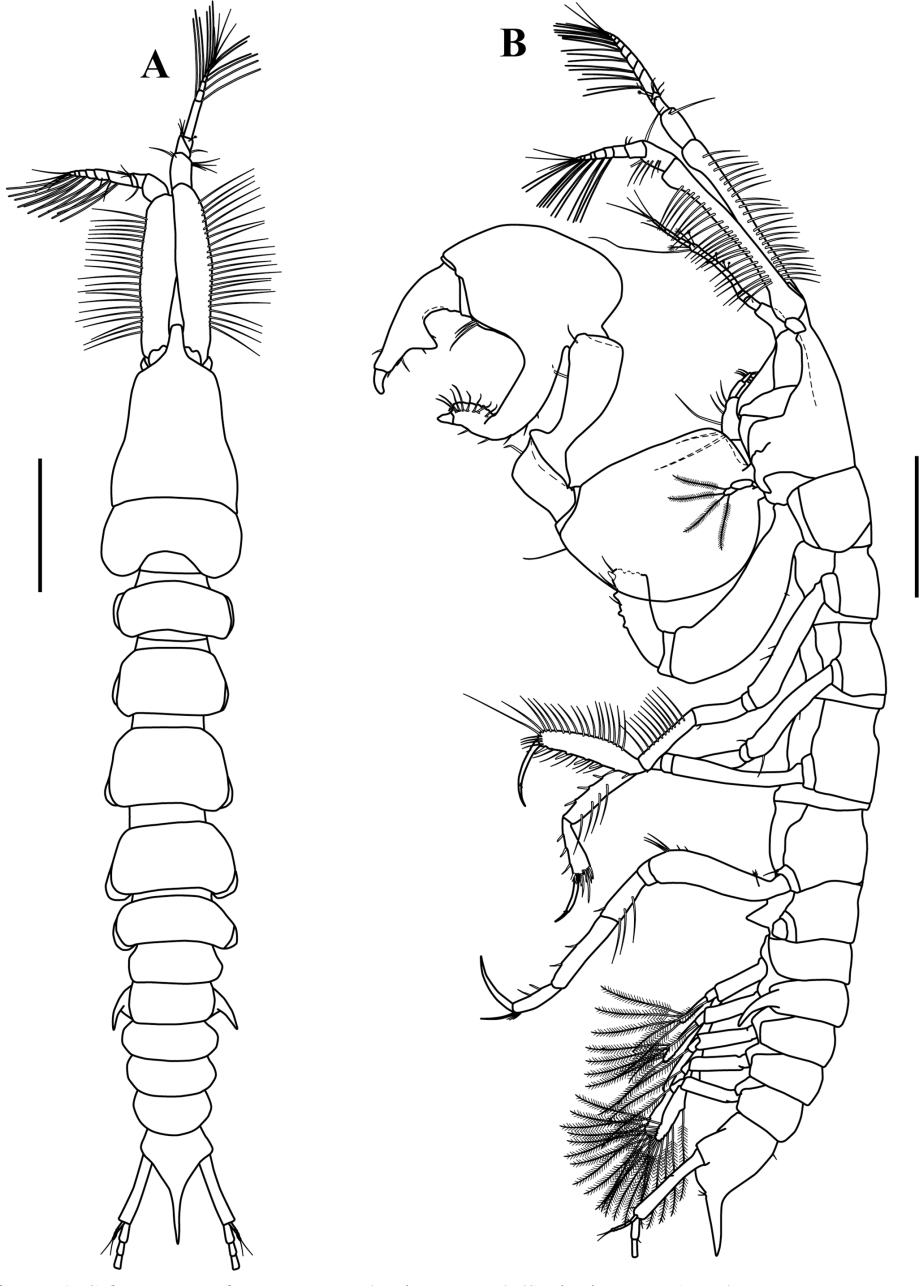
*Sphyrapus caribensis* sp. nov., paratype male. (A) Dorsal view; (B) lateral view. Scale bars = 1.0 mm.

**Figure 13:**
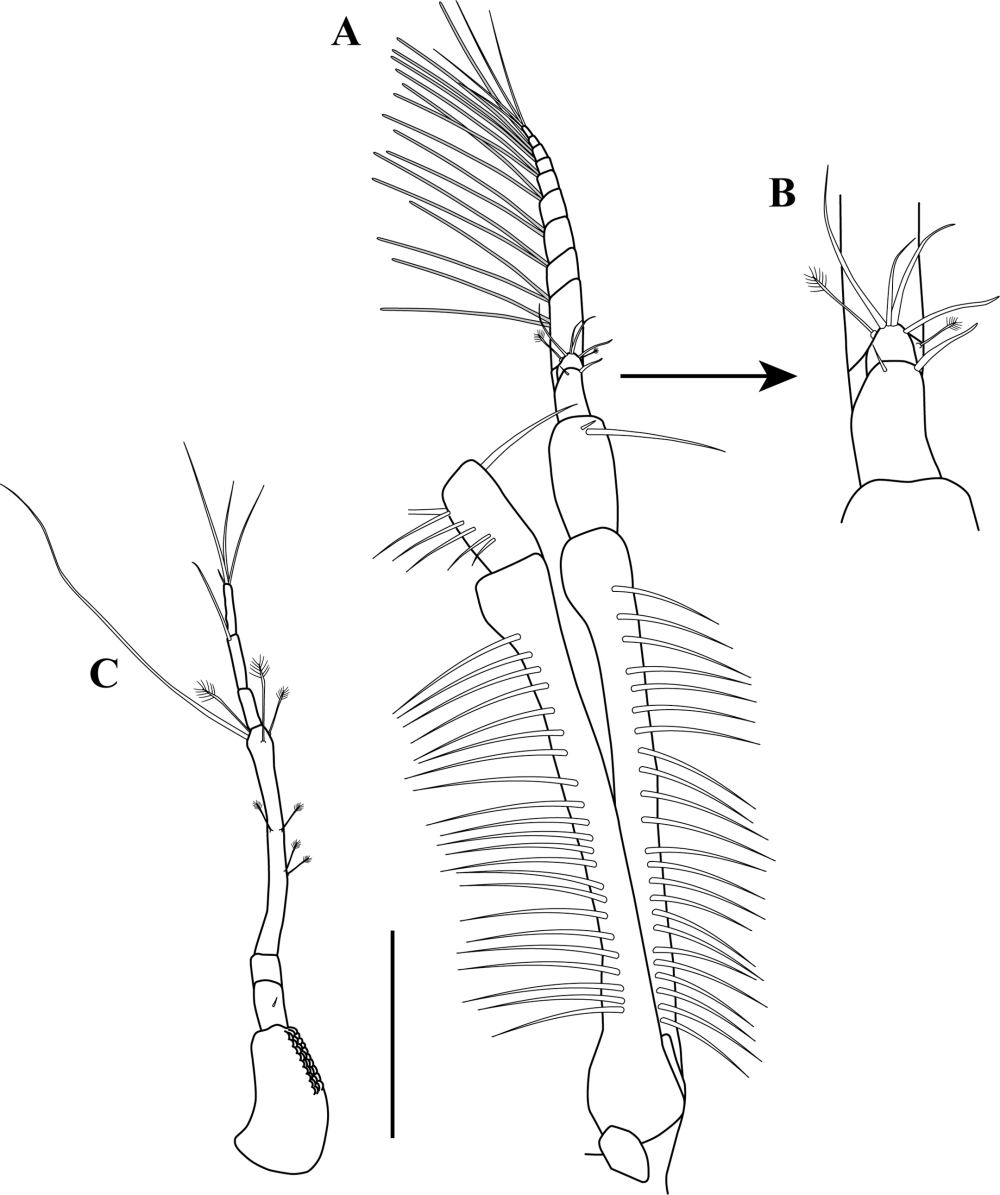
*Sphyrapus caribensis* sp. nov., paratype male. (A) Antennule, outer view; (B) enlargement of accessory flagellum; (C) antenna, outer view. Scale bar = 1.0 mm for A, C.

**Figure 14:**
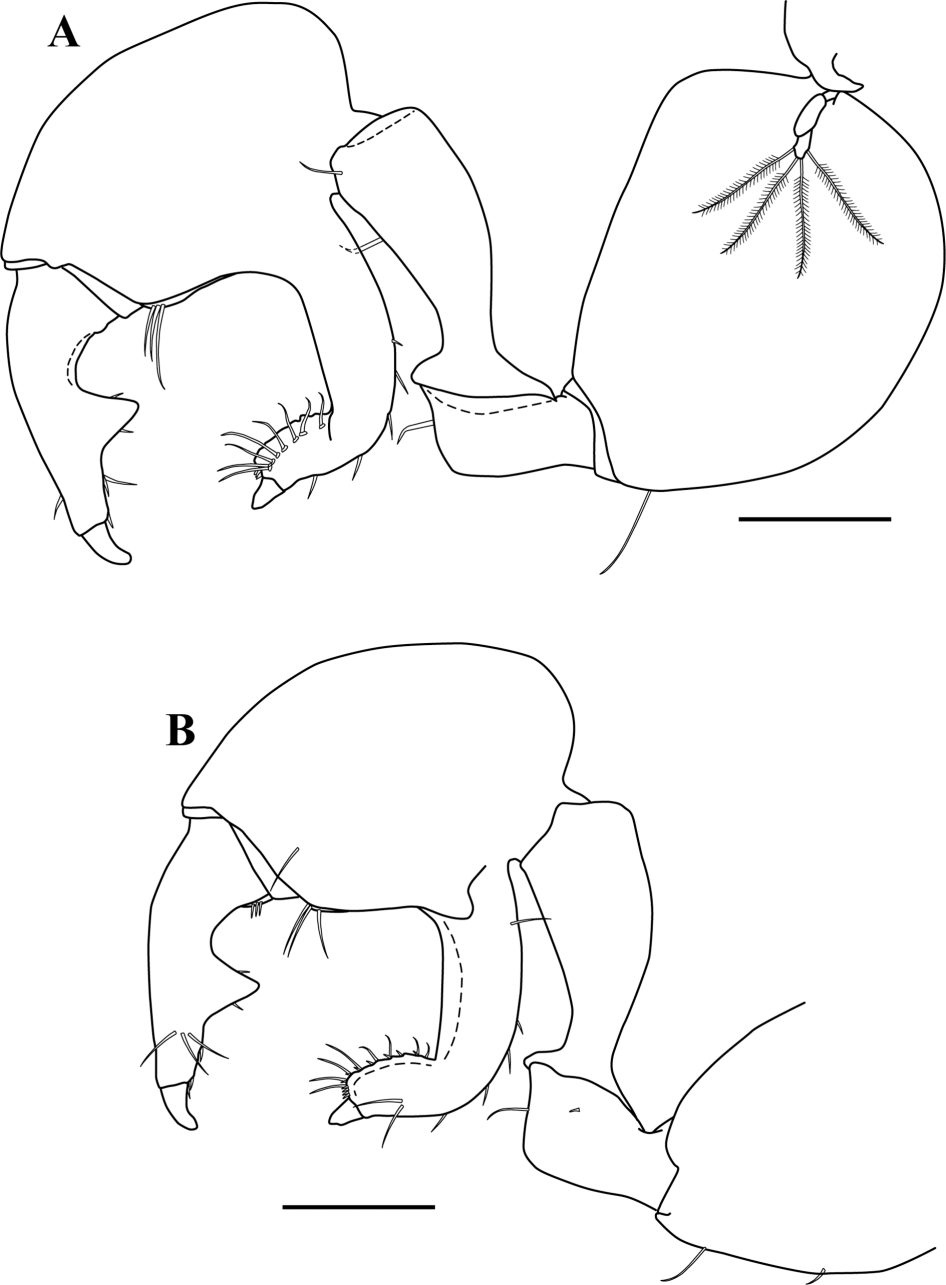
*Sphyrapus caribensis* sp. nov., paratype male. (A) Right cheliped, outer view; (B) right cheliped, inner view. Scale bars = 0.5 mm.

**Figure 15:**
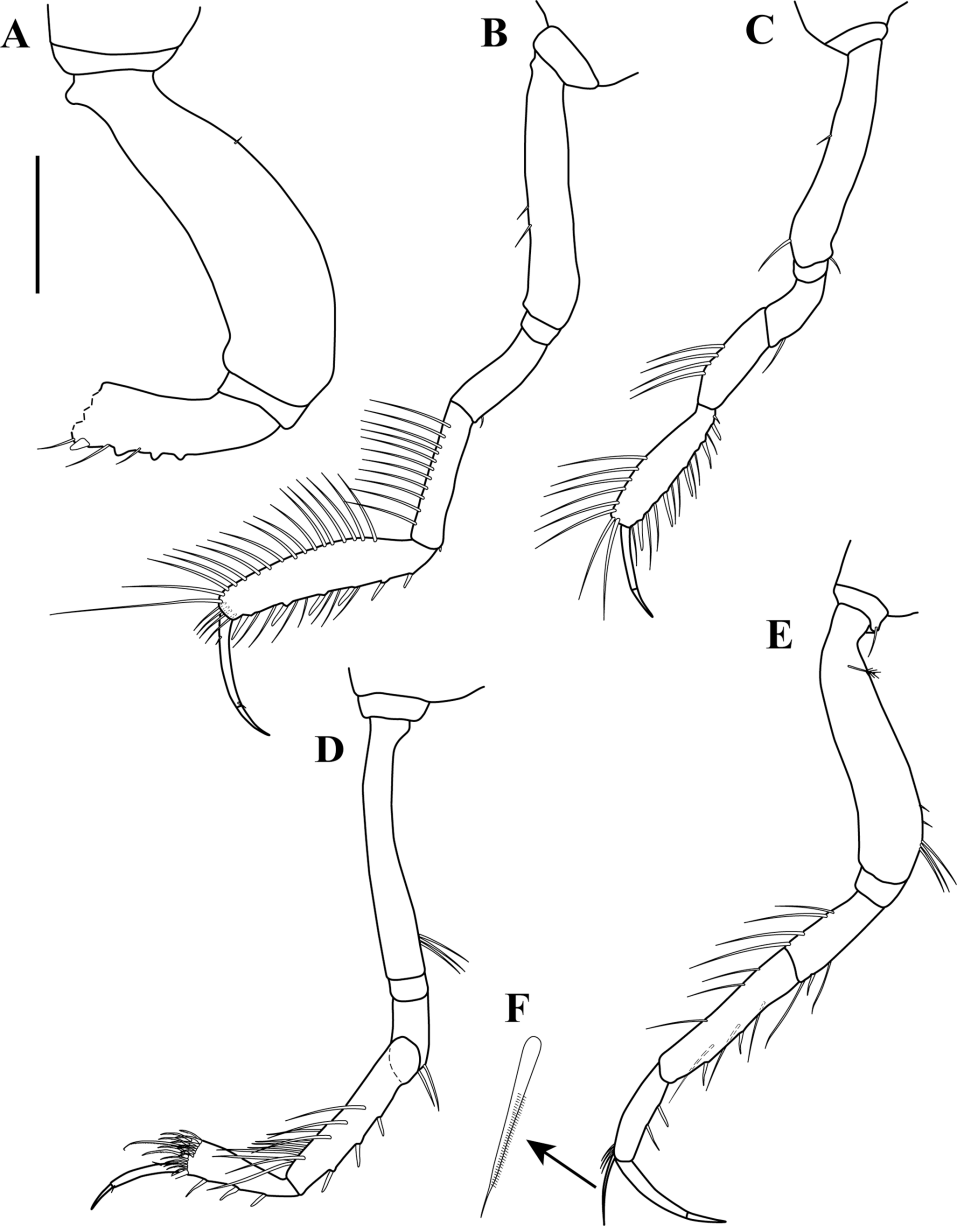
*Sphyrapus caribensis* sp. nov., paratype male. (A) Pereopod-1; (B) pereopod-2; (C) pereopod-3; (D) pereopod-4; (E) pereopod-5; (F) enlargement of strong bipinnate seta. Scale bar = 0.1 mm for A–E.

**Figure 16:**
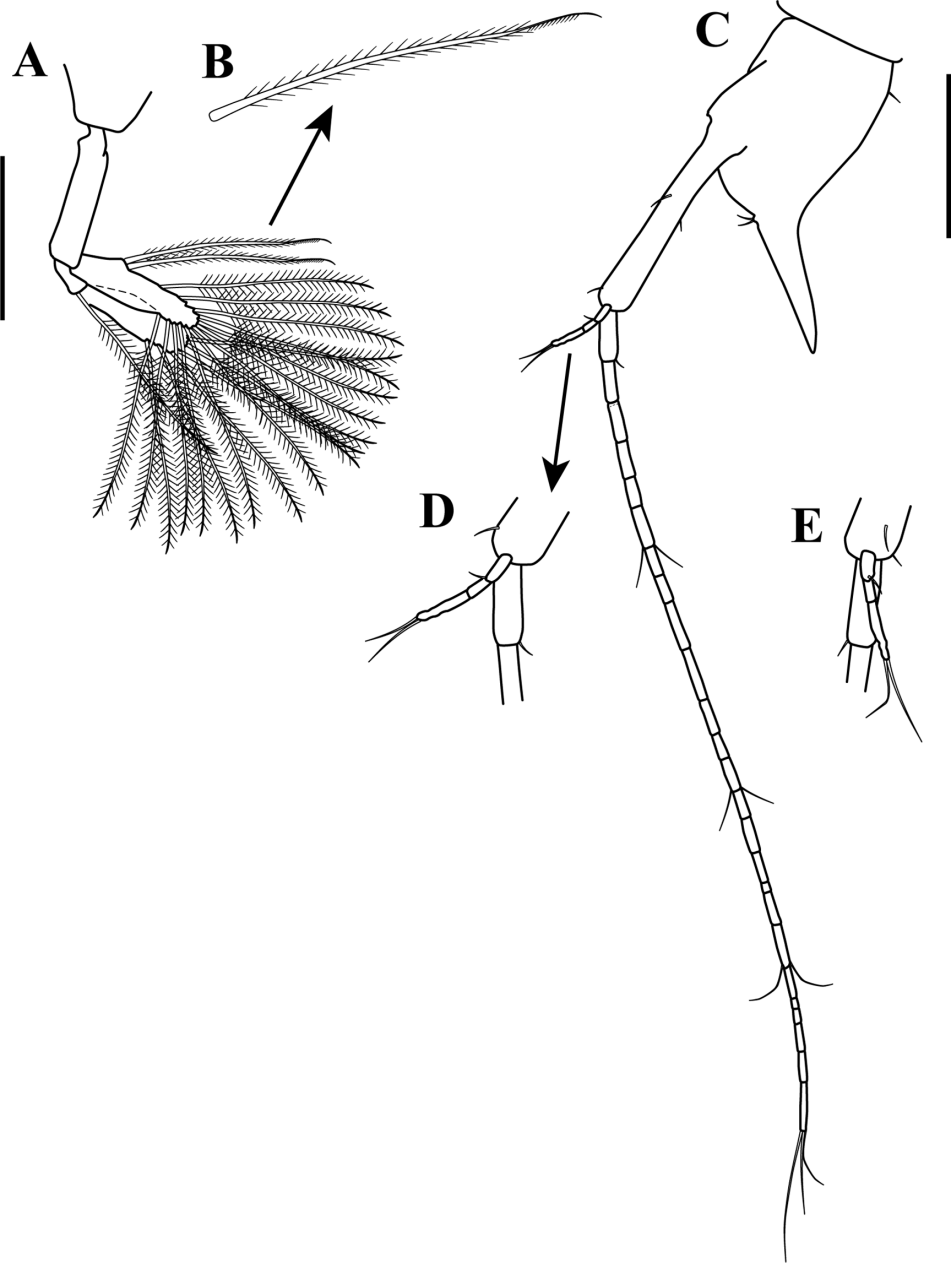
*Sphyrapus caribensis* sp. nov., paratype male. (A) Pleopod; (B) enlargement of plumose setae, attenuated distally into single serrate filament; (C) uropod; (D) enlargement of left exopod; (E) enlargement of right exopod. Scale bars = 0.1 mm for A, C.

**Figure 17:**
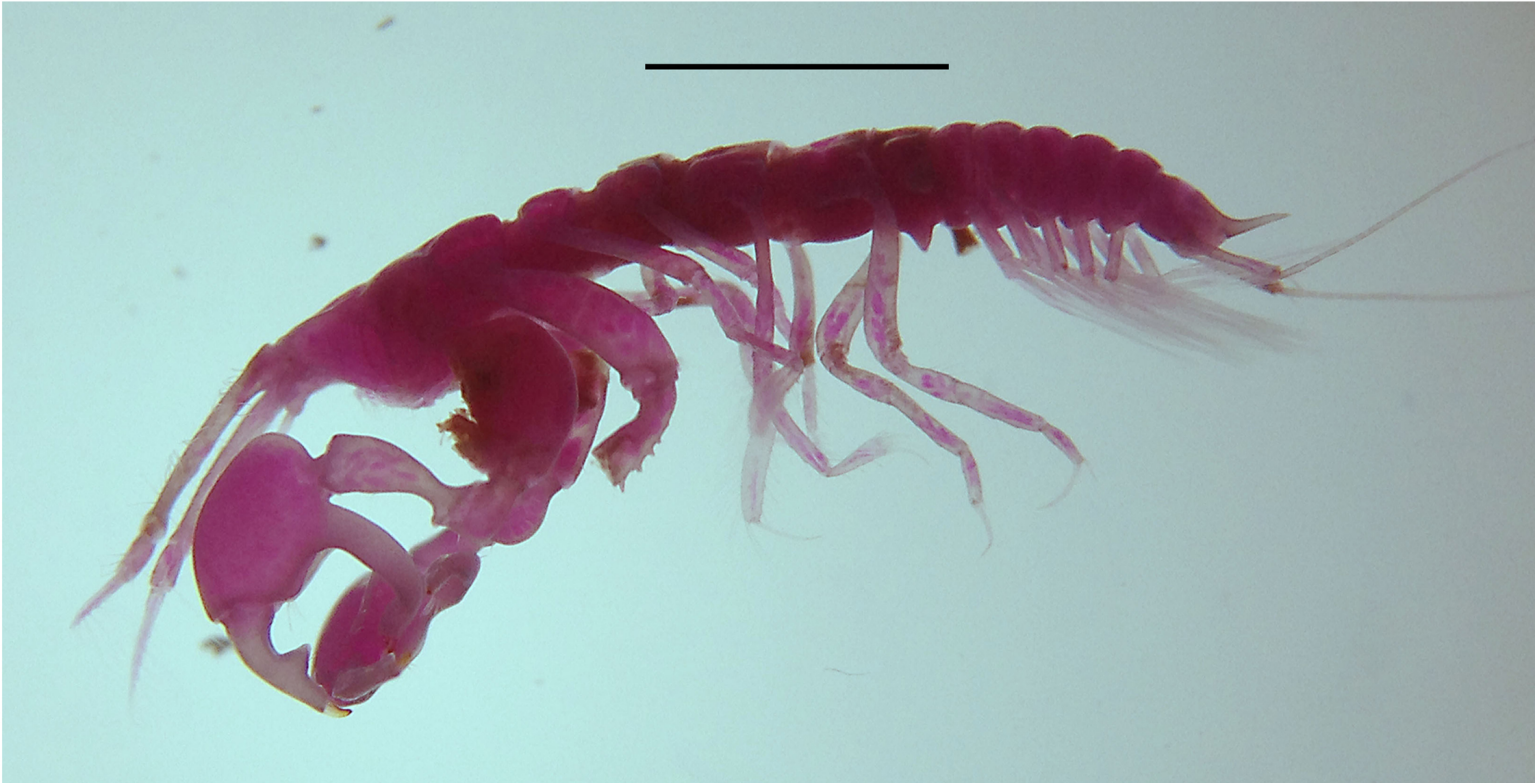
Digital image of *Sphyrapus caribensis* sp. nov., paratype male. Lateral view of habitus. Scale bar = 2.0 mm.

**Material examined. Holotype:** Non-ovigerous female (CBUMAG: MAC: 00001), TL 2.9 mm, Station (Stn) E13 Col5c (9°55′53.50″N–76°47′19.13″W), depth 2,945 m, substrata: “muddy bottom,” 03 May 2015.

**Paratypes:** One adult male (CBUMAG: MAC: 00002), TL 7.2 mm, Stn E15PAC (10°22′0.2″N–76°27′5.17″W), depth 2,821 m, substrata: “muddy bottom,” 26 July 2014; one non-ovigerous ♀ (dissected) (CBUMAG: MAC: 00006), length 2.7 mm, Stan E12Col5 (9°46′52.69″N–76°57′37.72″W), depth 3,010 m, substrata: “muddy bottom,” 01 May 2015.

**Additional material:** One ♀ (fragments), Stn E14Col5 (10°7′26.68″N–76°47′40.39″W), depth 3094 m, substrata: “muddy bottom,” 03 May 2015.

**Diagnosis:** *Female. Antennule* with peduncle having four articles; inner flagellum reduced to a tubercle. *Antenna* with seven articles. Right mandible with lacinia mobilis tridentate. *Maxillule* with eleven distal spiniform setae. *Labium* with palp having two distal spiniform setae. *Maxilliped* basis asetose. *Pereopods* 1–6 with basis having one seta on distoventral margin (i.e., simple seta on pereopods 1–4 and plumose seta on pereopods 5–6, respectively). *Pleopodal* exopod shorter than endopod. *Uropodal* exopod with three articles, longer than endopodal article-1.

*Male*: *Antennule* with peduncle having four articles; inner flagellum reduced to a tubercle; outer flagellum with nine articles. *Antenna* with seven articles. *Cheliped* forcipate with dactylus longer than fixed finger, with large, well-developed tooth on mid-ventral margin, tooth bearing small simple seta at each mid-outer margin. *Pleopod* endopod sub-equal in length to exopod.

**Etymology:** Named after the Spanish word “*Caribe,*” name of the area where this species was found.

**Type locality:** Offshore waters of Córdoba department (9°55′53.50″N–76°47′19.13″W), Colombia, South America.

**Distribution:** Colombian Caribbean at depths ranging from 2,818 to 2,945 m.

**Description:** Based on non-ovigerous female.

*Body* (Fig. 5) length 2.9 mm, slender, about 5.3 times as long as wide.
*Cephalothorax* (Figs. 5A and 5B) about 21% of TL, shorter than combined lengths of pereonites 1–3, length 1.1 times longer than wide, sub-rectangular, carapace asetose; anterior margin with conspicuous rounded rostrum; eyes-lobes present (small) without visual elements.
*Pereon* (Fig. 5A) about 48% of TL, pereonite-1 fused to cephalothorax (division line visible), nearly 1/3 as long as cephalothorax, laterally rounded, asetose (Fig. 5B); five free pereonites with lateral margins convex, asetose; pereonite-2 slightly shorter than pereonite-1, narrower; pereonite-3 longer than pereonite-2; pereonite-4 longest, sub-rectangular; pereonite-5 shorter than pereonite-4, but wider than other pereonites; pereonite-6 shortest.
*Pleon* (Fig. 5A) about 16% of TL, combined lengths of pleonites 1–5 shorter than pereonites 4–5 combined; all pleonites sub-equal in length, wider than long, bearing pleopods; pleonites-1, 3–5 laterally rounded; pleonite-2 with curved, sharp lateral apophysis.
*Pleotelson* (Figs. 5A and 11B) about 14% of TL; about same length as pleonites 3–5 combined, laterally expanded at attachment of uropods and with long distal spine same length as pleotelson.
*Antennule* (Figs. 5A, 6A–6C) peduncle with four articles, distinct, unfused. Article-1, 2.9 times as long as wide, inner margin with a proximal cluster of setules and row of 12 simple setae; outer margin with two broom-setae and row of 12 simple setae. Article-2, 1.4 times as long as wide; with two simple setae (one long and one short) on inner margin; with ventral row of five broom-setae; with three sub-distal simple setae on outer margin. Article-3 wider than long, with two inner distal simple setae; with outer distal long simple seta. Article-4, sub-equal in length to article-3, with one broom-seta. Outer flagellum with five articles: article-1, 4.3 times as long as wide, with distoventral convoluted aesthetasc (Fig. 6C); article-2, 1.4 times as long as wide, with distoventral simple seta and convoluted aesthetasc. Articles 3–5, sub-equal in length, articles 3–4 with distal setae and convoluted aesthetasc, article-5 with four simple setae of various lengths. Inner flagellum uniarticulate, as long as broad, with one broom-seta and four distal simple setae of various lengths (Fig. 6B).
*Antenna* (Figs. 6D and 6E) with seven articles; article-1 with outer expansion and with crenulate distodorsal margin, distally setulose; article-2, 1.3 times as long as wide, with oblique articulation with article-3, with simple setae on mid-ventral margin; article-3, 1.5 times as wide as long, asetose; article-4 elongate, 5.6 times as long as wide, dorsal margin with three broom-setae (one in the middle and two sub-distal), outer margin with two (one sub-distal) broom-setae and one sub-distal long (i.e., longer than the three latest articles combined) simple seta, ventral margin with three broom-setae; article-5, 1.5 times as long as wide; article-6, 2.1 times as long as wide, with distoventral simple seta; article-7, 3.3 times as long as wide, with three simple setae of various lengths.
*Mouthparts. Labrum* not recovered. *Mandibles* (Figs. 7A–7D) left mandible, with incisor with five uneven denticles, lacinia mobilis broad with four denticles, setiferous lobe with seven multi-furcate setae (Fig. 7A). Right mandible incisor with two uneven denticles, lacinia mobilis tridentate (Fig. 7C), setiferous lobe with four multi-furcate setae (Fig. 7B). Molar process of left and right mandibles similar, with grinding surface having well-developed micro-denticles and plumose marginal setae (Fig. 7D).
*Maxillule* (Fig. 7E) inner endite with five setulate distal setae, outer margin with several short rows of setules. Outer endite with eleven distal spiniform setae and two sub-distal setulose setae, outer margin with several short rows of setules; palp biarticulate with five setae and one hook-tipped seta sub-distally.
*Maxilla* (Figs. 8A–8F) inner margin with four spines, a row of two to four small simple setae, microtrichia, and two simple setae (Fig. 8A); outer lobe of moveable endite, with three finely inner pinnate-outer setulose spiniform setae (Fig. 8B) and three inner pinnate spiniform setae (Fig. 8C); inner lobe of moveable endite five simple setae and with four inner pinnate-plumose spiniform setae (Fig. 8D); outer lobe of fixed endite with two simple setae, four trifurcate-plumose spiniform setae (Fig. 8E), and one short bipinnate seta; inner lobe of fixed endite with four inner pinnate spiniform setae (Fig. 8F), and row of ∼31 basally swollen setae.
*Labium* (Figs. 8G–8H) inner margin setulose. Palp setulose with two distal spiniform setae (Fig. 8H).
*Maxilliped* (Figs. 8I and 8J) basis wider than longer, asetose (Fig. 8I). *Palp*: article-1, outer margin with small simple seta; inner sub-distal margin with long plumose seta. Article-2, 1.5 times as long as wide, outer margin with distal spiniform seta; inner margin with sub-proximal very long plumose seta and ten (one broken) simple setae of varying lengths. Article-3 outer margin asetose; inner margin with three strongly developed simple setae, and two (one short and one long) simple setae. Article-4 with five strongly developed setulate setae (Fig. 8J) on inner margin and three (one short and two long) distal setae. Endite setulose (Fig. 8K), with inner margin having two coupling hooks (Fig. 8I), row of six to eight basally swollen setulate setae (Fig. 8L), one bipinnate spiniform setae (Fig. 8M), two inner setulate-outer plumose spiniform setae (Fig. 8N), a cluster of three to four apically bidentate or grooved spiniform setae with outer setulose margin (Fig. 8O), and one long outer setulose seta.
*Epignath* not recovered.
*Cheliped* (Figs. 9A–9D) hammer-like. Basis, 1.2 times as long as wide, with small simple seta on mid-ventral margin and long sub-distal ventral simple seta. Merus subrectangular, with sub-distal ventral simple seta. Carpus, 1.3 times as long as wide, with one sub-distal simple seta on dorsal margin; with two sub-distal simple setae on ventral margin. Propodus massive, 1.2 times as long as wide; with two simple setae on outer-medial margin near articulation of dactylus, crenulate dorsal margin of fixed finger (Fig. 9B), with row of five to six sub-marginal simple setae on outer incisive margin, with two ventral setae, claw robust. Dactylus longer than fixed finger, unguis robust. *Inner surface* (Fig. 9C): Merus with small simple seta on sub-distal ventral margin. Propodus with small simple seta on dorsal margin, with two simple setae near articulation of dactylus; fixed finger with two sub-distal ventro-inner spiniform setae (Fig. 9D). Dactylus with three simple setae on sub-distal margin. Exopod with three articles, article-3 bearing four plumose setae.
*Pereopod*-*1* (Fig. 10A) distinctly larger and longer than other five pereopods. Basis stout, 2.9 times as long as wide; sub-proximal dorsal margin with broom-seta; distoventral margin with one simple seta. Ischium wider than long, with simple seta on mid-ventral margin. Merus, 2.2 times as long as wide; dorsal margin with sub-distal outer simple seta; ventral margin with four simple setae and distoventral strong spiniform seta, with sub-distal outer simple setae. Carpus, 3.3 times as long as wide; dorsal margin with three simple setae and sub-distal cluster of two to three simple setae of unequal lengths; ventral margin with five strong spiniform setae, distal-most largest, and two simple setae. Propodus, 2.3 times as long as wide; dorsal margin with row of 13 simple setae; ventral margin with four strong spiniform setae becoming longer distally, with two to three denticles before each strong spiniform setae, with small sub-distal simple seta; inner margin with one simple seta near articulation of dactylus. Dactylus elongate, curved, together with unguis shorter than propodus; dactylus longer than unguis. Exopod with three articles; article-1 very small, article-3 with five plumose setae.
*Pereopod-2* (Fig. 10B) shorter and more gracile than pereopod-1. Basis, 4.8 times as long as wide; with sub-proximal outer broom-seta; ventral margin with sub-proximal broom-seta and one long distoventral simple seta. Ischium wider than long, with simple seta on mid-ventral margin. Merus, 2.0 times as long as wide, ventral margin with sub-distal simple seta and sub-distal outer simple seta. Carpus, 1.7 times as long as wide; dorsal margin with row of four to six simple setae; ventral margin with row of three simple setae. Propodus, 3.0 times as long as wide; dorsal margin with row of 11–12 simple setae; ventral margin with five small spiniform setae and three simple setae; inner margin with three distal simple setae near articulation of dactylus. Dactylus elongate, curved, together with unguis shorter than propodus; dactylus longer than unguis.
*Pereopod-3* (Fig. 10C) similar in form to pereopod-2, but shorter. Basis without sub-proximal broom-seta. Carpus with dorsal margin having a row of three simple setae; ventral margin asetose. Propodus with dorsal margin having a row of six simple setae; ventral margin with four small spiniform setae and two simple setae.
*Pereopod-4* (Figs. 10D and 10G–10J) basis, 6.3 times as long as wide; dorsal margin with two (one sub-proximal and one on mid-margin) broom-setae (Fig. 10G); ventral margin with small sub-distal simple seta and distoventral simple seta. Ischium wider than long, with distoventral simple seta. Merus, 1.9 times as long as wide, ventral margin with two sub-distal simple setae. Carpus, 4.0 times as long as wide; dorsal margin with a cluster of three sub-distal bipinnate (difficult to observe, even in high magnification) setae of varying lengths (Fig. 10H); outer margin with a row of four bipinnate setae; ventral margin with three small spiniform setae. Propodus, 3.3 times as long as wide; dorsal margin with row of four sub-distal short serratopinnate setae (Fig. 10I), distally with crown of 17 (eight of them on inner view (Fig. 10J)) long serratopinnate setae of varying lengths, with one bipinnate seta, longer than dactylus and unguis combined; ventral margin with three spiniform setae. Dactylus elongate, curved, together with unguis shorter than propodus; dactylus longer than unguis. Exopod on sub-proximal dorsal margin, vestigial.
*Pereopod-5* (Figs. 10E and 10K) basis, 4.1 times as long as wide; dorsal margin with two (one sub-proximal and one on mid-margin) broom-setae; outer margin with broom-seta; ventral margin with small sub-distal simple seta and distoventral plumose seta. Ischium wider than long, with mid-ventral simple seta. Merus, 2.1 times as long as wide, dorsal margin with two sub-distal simple setae; ventral margin with three plumose setae and sub-distal simple seta. Carpus, 3.2 times as long as wide; dorsal margin with two bipinnate setae; sub-distal outer margin with bipinnate seta; ventral margin with three bipinnate setae of unequal lengths and two small spiniform setae. Propodus, 2.7 times as long as wide; inner view with row of 13 short serratopinnate setae (Figs. 10E and 10K) becoming longer distally, with one strong bipinnate seta longer than dactylus and unguis combined; mid-ventral outer view with simple seta. Dactylus elongate, curved, together with unguis longer than propodus; dactylus longer than unguis. Exopod on sub-proximal dorsal margin, vestigial.
*Pereopod-6* (Figs. 10F and 10L) similar in form to pereopod-5, shorter than other five pereopods. Basis with two mid-outer broom-setae. Merus with dorsal margin with two sub-distal plumose setae; ventral margin with sub-distal plumose seta. Carpus with dorsal margin having one bipinnate seta. Propodus with inner view having a row of seven short serratopinnate setae (Fig. 10L). Dactylus and unguis with combined length slightly shorter than propodus. Exopod absent.
*Pleopods* (Fig. 11A) five similar, well-developed, biramous pairs. Basal article broad, shorter than both rami, with plumose seta on distal inner margin. Exopod shorter than endopod, with proximal article bearing plumose seta on distal outer margin, distal article with six distal plumose setae. Endopod with one or two plumose setae on mid-inner margin, attenuated distally into single filament, with six plumose setae on distal margin.
*Uropod* (Fig. 11B) biramous. Basal article, 4.7 times as long as wide, with sub-distal simple setae on outer margin. Exopod of three articles, longer than endopodal article-1; article-1 sub-equal length that of article-2, with simple seta on sub-distal outer margin; article-2 asetose; article-3 longer than article 1 and 2 combined, with two distal simple setae. Endopod elongate, with 9–10 articles; articles bearing broom-setae or simple seta, or both; terminal article with two broom-setae and four simple distal setae of unequal lengths.
*Adult male*. Based on paratype (CBUMAG: MAC: 00002). *Body* (Figs. 12A, 12B and 17) slender, about 7.4 times as long as wide.
*Cephalothorax* (Fig. 12) about 20% of TL, shorter than combined lengths of pereonites 1–3, length 1.4 times longer than wide, sub-rectangular, carapace asetose; anterior margin with long rostrum semi-acute; eye-lobes present without visual elements.
*Pereon* (Fig. 12) about 50% of TL; pereonite-1 fused to cephalothorax (divisor line visible), 1/3 as long as cephalothorax, laterally rounded, asetose; five free pereonites with lateral margins convex, asetose; pereonite-2 slightly shorter than pereonite-1; pereonites 3 and 5 sub-equal in length, slightly longer than pereonites 1–2; pereonite-4 slightly longer and wider than others; pereonite-6 shortest.
*Pleon* (Fig. 12) about 20% of TL; combined lengths of pleonites 1–5 shorter than pereonites 4–6 combined; all pleonites sub-equal, wider than long, bearing pleopods; pleonite-2 with curved, sharp lateral spine-like apophysis; pleonites 3–5 laterally rounded (Fig. 12B).
*Pleotelson* (Fig. 12) about 10% of TL; about same length as pleonites 3–5 combined, laterally expanded at attachment of uropods and with long distal spine.
*Antennule* (Figs. 13A and 13B) with four peduncular, distinct, unfused articles. Article-1, 14.0 times as long as wide, with distoventral lobe (visible only in outer view), with row of 23 simple setae along the inner and outer margin. Article-2, twice as long as wide, distodorsal margin with two (one long and one short) simple setae, with row of six (one broke) oblique simple setae, distal largest. Article-3, 1.6 times as long as wide, with simple setae on distodorsal margin and one small simple seta on distal mid-outer margin. Article-4, wider than long, with one broom-seta. Outer flagellum with eight articles: article-1, 2.5 times as long as wide, with two (one in the middle and one distally) cluster of two to three aesthetascs; articles 2–7 with distal cluster of two to three aesthetascs; article-8 minute, terminating in three simple setae of varying length. Accessory flagellum uniarticulate, with one broom-seta and four distal simple setae of various lengths (Fig. 13B).
*Antenna* (Fig. 13C) with seven articles, squama absent. Article-1 with outer expansion with crenulate distal margin, asetose. Article-2, 1.7 times as long as wide, with simple seta on mid-outer margin. Article-3 slightly wider than long, asetose. Article-4 elongate, 14.1 times as long as wide, dorsal margin with six broom-setae, three near to the mid-margin and three sub-distally, ventral margin with one broom-seta near to mid-margin and one very long distal seta. Article-5, 3.1 times as long as wide, asetose. Article-6, 4.7 times as long as wide, ventral margin with long distal seta, longer than article-7. Article-7 sub-equal length that of article-6, terminating in four simple setae of varying length.
*Mouthparts* (not illustrated), similar to female.
*Cheliped* (Figs. 12B, 14A and 14B) dimorphic, massive, proportionately larger than in female. Basis, 1.3 times as long as wide, with long sub-distal ventral simple seta. Merus subrectangular, with sub-distal ventral simple seta. Carpus, 3.0 times as long as wide, curved, wider distally, with ventral proximal apophysis, with two simple setae on ventral margin. Chela forcipate; fixed finger with rectangular distal part. Propodus arcuate, 1.2 times as long as wide, with cluster of three simple setae on sub-distal ventral margin; fixed finger with crenulate incisive margin, with row of eight sub-marginal simple setae on outer incisive margin, with five ventral setae, claw robust. Dactylus longer than fixed finger, with large, well-developed tooth on mid-ventral margin; tooth bearing small simple seta at each mid-outer margin, with two (one sub-distal and one distal) spines on ventral margin, unguis robust. *Inner surface* (Fig. 14B): basis with small spiniform seta on mid-ventral margin. Merus with small simple setae on sub-distal ventral margin. Propodus with simple seta near articulation of dactylus; fixed finger with simple seta on ventro-proximal margin, with two sub-distal ventral setae, with row of eleven spiniform setae on dorsal margin. Dactylus with cluster of three small spiniform setae on ventro-proximal margin, with three simple setae on sub-distal margin. Exopod with three articles, article-3 bearing four plumose setae.
*Pereopod-1* (broken, partially illustrated) (Fig. 15A) basis, 3.2 times as long as wide, dorsal margin with proximal apophysis, sub-proximal ventral margin with small seta. Ischium wider than long, asetose. Merus (broken), ventral margin with three denticles, with three simple setae and one spiniform seta.
*Pereopod-2* (Fig. 15B) basis, 5.7 times as long as wide, with two simple setae near to the mid-dorsal margin. Ischium wider than long, asetose. Merus, 2.7 times as long as wide, with small simple seta on sub-distal ventral margin. Carpus, 5.0 times as long as wide; dorsal margin with a row of 11 simple setae; distoventral margin with a small spiniform seta. Propodus, 4.5 times as long as wide; dorsal margin with a row of 17 simple setae; ventral margin with six spiniform setae and seven simple setae; inner margin with a row of four simple setae. Dactylus elongate, curved, together with unguis shorter than propodus; dorsal margin with proximal small seta; distoventral margin with small seta; dactylus longer than unguis.
*Pereopod-3* (Fig. 15C) similar in form to pereopod-2, but shorter. Basis, 6.1 times as long as wide; dorsal margin with two (one in the middle and one sub-distally) simple setae; distoventral margin with simple setae. Merus, 2.0 times as long as wide, with simple seta on sub-distal ventral margin. Carpus, 2.7 times as long as wide, dorsal margin with a row of four simple setae. Propodus, 3.7 times as long as wide; dorsal margin with a row of eight simple setae; ventral margin with six (one small) spiniform setae and eight simple setae.
*Pereopod-4* (Fig. 15D) basis, 7.5 times as long as wide; ventral margin with a cluster of three sub-distal simple setae. Ischium wider than long, asetose. Merus, 2.1 times as long as wide; ventral margin with two sub-distal simple setae. Carpus, 5.2 times as long as wide; dorso-outer and inner margin with a row of four and seven simple setae of varying lengths, respectively; ventral margin with three spiniform setae. Propodus, 3.5 times as long as wide; dorsal margin with a row of seven sub-distal serratopinnate setae, distally with crown of 24 (13 of them on inner view) serratopinnate setae of varying lengths, with one bipinnate seta shorter than dactylus; ventral margin with four (one broken) spiniform setae. Dactylus elongate, curved, together with unguis shorter than propodus; dorsal margin with proximal small seta; distoventral margin with a small seta; dactylus longer than unguis. Exopodite absent.
*Pereopod-5* (Figs. 15E and 15F) basis, 5.0 times as long as wide; sub-proximal outer margin with broom-seta: ventral margin with two small simple setae and a cluster of three setae, sub-distally. Ischium wider than long, asetose. Merus, 2.5 times as long as wide; dorsal margin with a row of four simple setae; sub-distal ventral margin with two simple setae and one spiniform seta. Carpus, 5.2 times as long as wide; dorsal margin with three simple setae; ventral margin with two spiniform setae and three simple setae. Propodus, 3.6 times as long as wide; inner view with a row of 14–15 short serratopinnate setae becoming longer distally (no illustrated), with one strong bipinnate seta (Fig. 15F) shorter than dactylus; ventral margin with two spiniform setae. Dactylus elongate, curved, together with unguis longer than propodus; dactylus longer than unguis. Exopodite absent.
*Pereopod-6* missing.
*Pleopods* (Figs. 16A and 16B) five similar, well-developed, biramous pairs, more slender than in female. Basal article same length as both rami, asetose. Exopod and endopod sub-equal in length, with proximal article bearing plumose seta on outer margin, distal article with 13–15 sub-distal and distal plumose setae. Endopod with two plumose setae on mid-inner margin, attenuated distally into a single filament (Fig. 16B) near to mid-inner margin, with 13–15 plumose setae on distal margin.
*Uropod* (Figs. 16C–16E) biramous; basis elongate, 8.3 times as long as wide, with inner and outer simple seta on mid and sub-distal margin. Exopod of three articles, longer than endopodal article-1; article-1 longer than article-2, with simple seta on distal outer margin; article-3 same length than article 1 and 2 combined, with two distal simple setae. Endopod elongate, with ∼25 articles; article-1 with simple setae on distoinner margin; articles 6, 13, and 19 with simple setae on disto-inner and outer margin; last article with three distal simple setae of unequal length.

### Ecological notes

Specimens of *Sphyrapus caribensis* sp. nov. (Fig. 17) were collected from muddy bottoms with a content of mud and clay between 93.7% and 98.2%. Other physicochemical parameters of the surrounding waters were a temperature of 4.1 °C, salinity of 35 ppm, pH of 7.96–8.0, and DO of 4.7–6.7 mg/L.

## Discussion

To date, only two species have been described in the genus *Sphyrapus*, *Sphyrapus malleolus* and *Sphyrapus meknes*; however, the male of *Sphyrapus meknes* is still unknown. The females of *Sphyrapus caribensis* sp. nov. appear to be most closely related to *Sphyrapus malleolus* from the northeast Atlantic (Norman & Stebbing, 1886; Błażewicz-Paszkowycz, Bamber & Cunha, 2011) in having pleonite-2 with curved, sharp lateral apophysis and the uropodal exopod being tri-articulate (*Sphyrapus malleolus sensu* Norman & Stebbing, 1886). However, the new species can be distinguished from *Sphyrapus malleolus* by (1) maxilliped basis without long distal seta (present in *Sphyrapus malleolus*), (2) pereopods 1 and 2 with basis bearing one simple seta on distoventral margin (cluster of four to five simple setae in *Sphyrapus malleolus*), (3) the presence of vestigial exopod (e.g., in non-ovigerous females) on pereopods 4 and 5 (absent in *Sphyrapus malleolus*), and (4) pleopod basis with one plumose seta (with three in *Sphyrapus malleolus*).

**Figure 18:**
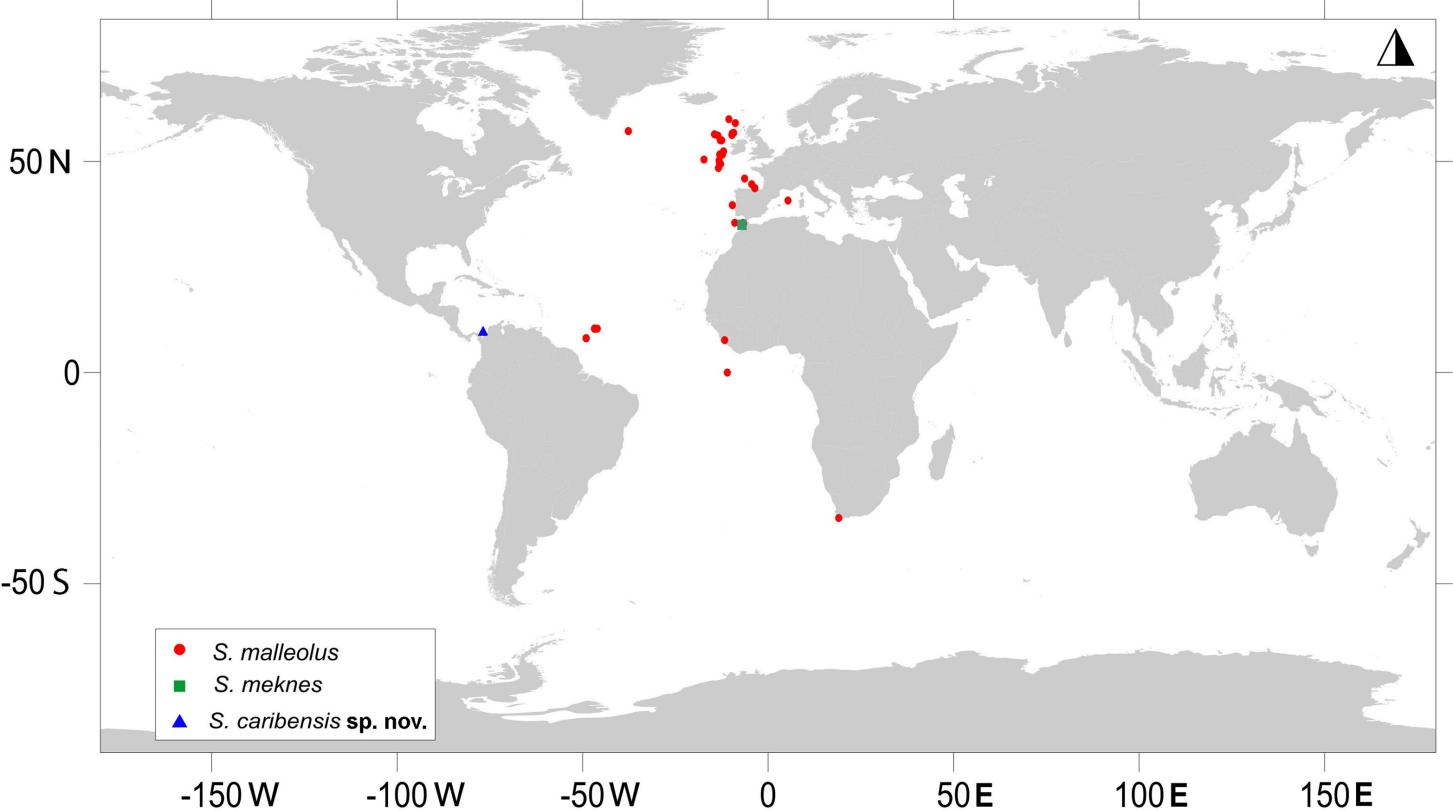
Map showing the worldwide distribution of *Sphyrapus*. *Sphyrapus malleolus* (red circles), *Sphyrapus meknes* (green square), *Sphyrapus caribensis* sp. nov. (blue triangle) [Data from: Norman & Stebbing (1886); Bonnier (1896); Lo Bianco (1903); Richardson (1905); Barnard (1920); Băcescu (1982); Holdich & Jones (1983); Băcescu (1984); Bamber (2000); Bird (2001); Błażewicz-Paszkowycz, Bamber & Cunha (2011); Natural History Museum (2014); Morales-Núñez et al. (this study)].

**Figure 19:**
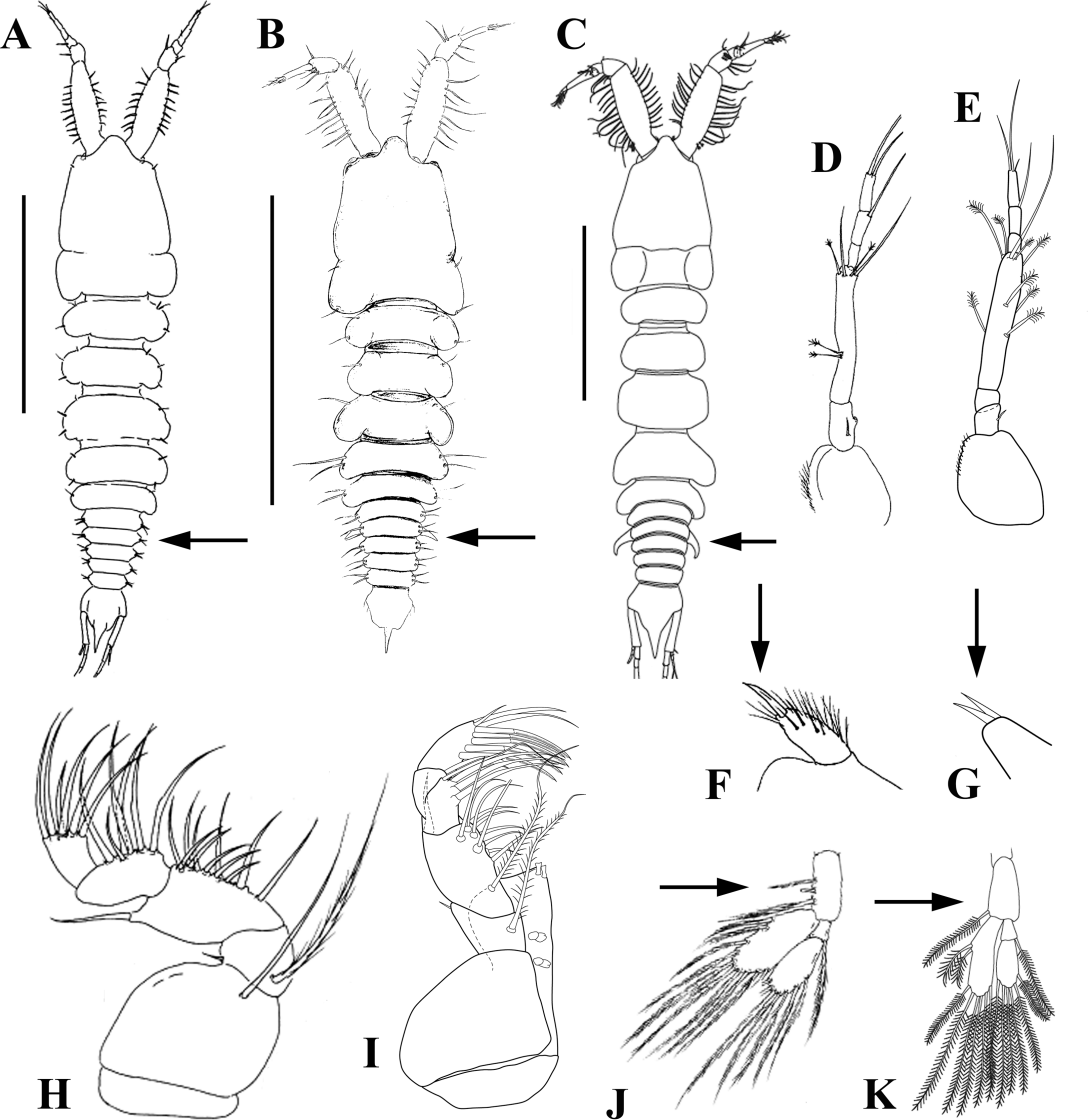
Female dorsal view. (A) *Sphyrapus meknes*; (B) *Sphyrapus malleolus*; (C) *Sphyrapus caribensis* sp. nov. Antenna; (D) *Sphyrapus meknes*; (E) *Sphyrapus caribensis* sp. nov. Labium; (F) *Sphyrapus malleolus*; (G) *Sphyrapus caribensis* sp. nov. Maxilliped; (H) *Sphyrapus malleolus*; (I) *Sphyrapus caribensis* sp. nov. Pleopod; (J) *Sphyrapus malleolus*; (K) *Sphyrapus caribensis* sp. nov. [Figures modified from: A, B, D, F, and H, Błażewicz-Paszkowycz, Bamber & Cunha (2011); C, E, G, and I, Morales-Núñez et al. (this study)]. Not to scale.

*Sphyrapus caribensis* also exhibits similarities with *Sphyrapus meknes* recently described from 703 m in waters of the Gulf of Cadiz (Northeast Atlantic) (Błażewicz-Paszkowycz, Bamber & Cunha, 2011). Both species share two unusual features: (1) the presence of plumose setae (=bifurcate-dendritic setae, Błażewicz-Paszkowycz, Bamber & Cunha, 2011) on pereopods 5–6 and (2) the presence of vestigial exopod on pereopods 4–5 in females (Błażewicz-Paszkowycz, Bamber & Cunha, 2011). Guţu & Heard (2002), Larsen (2005), and Drumm & Heard (2011) observed and reported the presence of exopods on pereopods 4 and 5 of the mancae within the apseudomorphan families Kalliapseudidae and Sphyrapodidae. Larwood (1954), however, has reported exopods on pereopods 4–6 in juveniles of a kalliapseudid *Cristapseudes omercooperi* (Larwood, 1954). Recently, Błażewicz-Paszkowycz, Bamber & Cunha (2011; p. 33) reported for the first time the presence of exopods on pereopods 4 and 5 in the adult female of *Sphyrapus meknes*. But, the illustrations and descriptions of the appendages of *Sphyrapus meknes* were based on a subadult female (p. 29; figs. 18–19, pp. 31–32, respectively), lacking its body length data. Thus, it is not clear whether or not the “adult female” in the study by Błażewicz-Paszkowycz, Bamber & Cunha (2011) actually corresponds to the brooding female of *Sphyrapus meknes*. Importantly, the females of *Sphyrapus malleolus* lack vestigial exopods on pereopods 4 and 5 (Błażewicz-Paszkowycz, Bamber & Cunha, 2011). The pereopod-6 of *Sphyrapus caribensis* sp. nov. seems to be less developed and far shorter than the pereopod-5, whereas both pereopods of *Sphyrapus malleolus* were subequal in length (Błażewicz-Paszkowycz, Bamber & Cunha, 2011), implying that our female specimens may be juveniles (we are unable to compare the lengths of those appendages with those in *Sphyrapus meknes* due to the lack of a scale bar for the pereopod-6). Re-examinations of the holotype of *Sphyrapus meknes* and observation of the brooding females of *Sphyrapus caribensis* in the future are needed to confirm whether brooding females of the two species have exopods on pereopods 4 and 5.

*Sphyrapus caribensis* can be differentiated from *Sphyrapus meknes* by (1) pleonite-2 with curved, sharp, lateral spine-like apophysis (absent in *Sphyrapus meknes*), (2) antennular inner flagellum present, one article (absent in *Sphyrapus meknes*), (3) antenna with seven articles (six in *Sphyrapus meknes*), (4) labium palp with two distal spiniform setae (one setulose spiniform seta in *Sphyrapus meknes*), and (5) uropodal exopod tri-articulate (two-articulate in *Sphyrapus meknes*).

The male of *Sphyrapus caribensis* is distinguishable from *Sphyrapus malleolus* by having (1) antennule flagellum with nine articles (five in *Sphyrapus malleolus*), (2) cheliped dactylus, with large, well-developed tooth on mid-ventral margin; tooth bearing small simple setae at each mid-outer margin (with four rounded apophyses along cutting edge in *Sphyrapus malleolus*), and (3) pleopod with exopod and endopod subequal in length (endopod longer than exopod in *Sphyrapus malleolus*).

The apparent lack of exopod in the pereopod-1 of the male of *Sphyrapus caribensis* might be an artefact due to the condition of the specimen, since the right pereopod-1 was missing and only a broken (i.e., basis to merus) left pereopod-1 was found.

It is important to highlight that *Sphyrapus malleolus* has a wide distribution (Fig. 18); however, tanaidaceans are considered to be an animal group showing more local distributions, especially, deep-water species (Larsen, 2005; Błażewicz-Paszkowycz, Bamber & Anderson, 2012). Therefore, we concur that a detailed morphological (and molecular) examination of specimens collected from various sites within its distribution must be conducted to determine if all of them are conspecific with *Sphyrapus malleolus* or rather undescribed species (Błażewicz-Paszkowycz, Bamber & Cunha, 2011). For instance, Błażewicz-Paszkowycz, Bamber & Cunha (2011) presented a satisfactory illustration of a specimen that they considered conspecific (i.e., morphologically consistent with the type-material) with *Sphyrapus malleolus*, based on material collected from Gulf of Cadiz. They stated however, that there are some subtle variations (e.g., peduncle of antennules and antennae) between the lectotype of *Sphyrapus malleolus* and their depicted specimen of *Sphyrapus malleolus*. Additionally, another noteworthy difference between these conspecific individuals is the presence of an uropodal exopod tri-articulate in *Sphyrapus malleolus sensu* Norman & Stebbing (1886) versus an uropodal exopod bi-articulate in *Sphyrapus malleolus sensu* Błażewicz-Paszkowycz, Bamber & Cunha (2011).

This is the first time that a member of the subfamily Sphyrapodinae has been reported and described from Colombian waters in the Caribbean Sea. There are only two previous records of this subfamily in the Caribbean Sea, *Sphyrapoides tuberculifrons* Guţu & Heard, 2002 from Grand Cayman Island (see Guţu & Heard, 2002) and *Sphyrapoides bicornis* Guţu & Iliffe, 1998 from the Bahamas (see Guţu & Iliffe, 1998). The occurrence of *Sphyrapus caribensis* sp. nov. in the deep marine waters of Colombia extends the distribution range of the genus *Sphyrapus* to the southern area of the Caribbean Sea (Fig. 18). The following key may be used to separate the species within the genus *Sphyrapus*.

**Key to the known species of *Sphyrapus* (females)**

1. Pleonite-2 **without** curved, sharp lateral spine-like apophysis (Fig. 19A). Antenna with six articles (Fig. 19D) *Sphyrapus meknes* [Northeast Atlantic: Gulf of Cadiz].
  —Pleonite-2 **with** curved, sharp lateral spine-like apophysis (Figs. 19B and 19C). Antenna with seven articles (Fig. 19E) 2
2. Labium with one distal spiniform seta (Fig. 19F). Maxillipedal basis with long distal seta (Fig. 19H). Pleopodal basis with three plumose setae (Fig. 19J) *Sphyrapus malleolus* [Greenland; Northeast Atlantic: from the Iceland Basin south to the Gulf of Cadiz,]
  —Labium with two distal spiniform setae (Fig. 19G). Maxillipedal basis without long distal seta (Fig. 19I). Pleopodal basis with one plumose seta (Fig. 19K) *Sphyrapus caribensis* sp. nov. [Northwest Atlantic: Colombian Caribbean]

## Supplemental Information

10.7717/peerj.3947/supp-1 Supplemental Information 1 Raw data–specimens of Spyrapodids used in this study. Geographical information, physicochemical data and specimens of Spyrapodids used in this study.

## Additional Information and Declarations

### Field Study Permissions

The following information was supplied relating to field study approvals (i.e., approving body and any reference numbers): Collection permits were granted by the National Authority of Environmental Licenses—ANLA. Permit numbers: FNA, ANLA No. 0723 de 2012; PAC, ANLA No. 0880 de 2014; COL5, ANLA No. 0440 de 2015.

### Data Availability

The following information was supplied regarding data availability: The raw data has been supplied as Supplemental Dataset Files.

### New Species Registration

The following information was supplied regarding the registration of a newly described species: *Sphyrapus caribensis* sp. nov. New taxon LSID: urn:lsid:zoobank.org:act:93557B1D-0BAE-458F-AFCC-CB6D07A90BEB Publication LSID: urn:lsid:zoobank.org:pub:2ADB8B48-19CB-4DF0-8FBD-CF9DA78E779D
